# Diet and female fertility: a population-based study re-evaluating the need for prescriptive dietary patterns

**DOI:** 10.3389/fnut.2025.1682549

**Published:** 2025-10-22

**Authors:** Simon Alesi, Jessica A. Grieger, Helena Teede, James R. Hébert, R. Kendal Moss, Sherry Price, Allison Hodge, Lisa Moran, Aya Mousa

**Affiliations:** ^1^Monash Centre for Health Research and Implementation, Monash University, Melbourne, VIC, Australia; ^2^Adelaide Medical School and Robinson Research Institute, The University of Adelaide, Adelaide, SA, Australia; ^3^Department of Epidemiology and Biostatistics, Cancer Prevention and Control Program, Arnold School of Public Health, University of South Carolina, Columbia, SC, United States; ^4^Department of Nutrition, Connecting Health Innovations LLC, Columbia, SC, United States; ^5^Cancer Epidemiology Division, Cancer Council Victoria, Melbourne, VIC, Australia; ^6^Centre for Epidemiology and Biostatistics, Melbourne School of Population and Global Health, The University of Melbourne, Parkville, VIC, Australia

**Keywords:** diet quality, energy-adjusted/dietary inflammatory index, mediterranean diet, fertility, infertility, diet, Australian Longitudinal Study on Women’s Health

## Abstract

**Introduction:**

Diet may impact female fertility via inflammatory pathways, but the value of specific anti-inflammatory dietary indices compared with general healthy eating guidelines is unclear. We examined associations between different measures of dietary inflammation and diet quality with female infertility in a large population-based study.

**Methods:**

Data for 5,489 participants from the Australian Longitudinal Study on Women’s Health were analysed (1973–1978 cohort assessed in 2009, at 31–36 years old; *n* = 1,289 fertility problems, *n* = 4,200 no fertility problems). Dietary inflammatory potential was assessed using the energy-adjusted dietary inflammatory index (E-DII™). Diet quality was examined using the dietary guideline index (DGI) and principal component analysis (PCA) for a posteriori patterns. Cross-sectional associations between these indices and self-reported fertility problems were assessed using logistic regression, adjusted for relevant covariates.

**Results:**

A diet with greater inflammatory potential was associated with higher odds of self-reported fertility problems (adjusted odds ratio (aOR) per 1-unit increase in E-DII: 1.13, 95% confidence interval (CI): 1.06, 1.19), with significant differences between the highest and lowest E-DII quartiles (aOR: 1.53, 95%CI: 1.23, 1.90). Higher dietary quality was associated with lower odds of self-reported fertility problems (aOR per 1-unit increase in DGI: 0.99, 95% CI: 0.99, 0.99), including when comparing highest and lowest DGI quartiles (aOR: 0.76, 95% CI: 0.61, 0.95). In PCA, consumption of a Mediterranean-style dietary pattern was associated with lower odds of self-reported fertility problems (aOR: 0.92, 95% CI: 0.88, 0.97), including when comparing highest and lowest quartiles (aOR: 0.70, 95% CI: 0.57, 0.85).

**Discussion:**

Our data suggest that following a generally healthy diet is associated with improved female fertility, whether by adherence to low inflammatory potential diets, Mediterranean-style dietary patterns or national dietary guidelines. These findings suggest that general, guideline-based healthy eating can support female fertility and may offer a flexible alternative to more prescriptive dietary approaches.

## 1 Introduction

Infertility, characterised by the inability to achieve clinical pregnancy after 12 months of regular unprotected intercourse (1), is a global health issue, impacting an estimated 48 million couples and 186 million individuals worldwide (2). Individuals with infertility often endure substantial emotional distress and a diminished quality of life, which adversely impact mental health and interpersonal relationships (3, 4). Beyond the psychosocial toll, infertility treatments such as in-vitro fertilisation (IVF) and assisted reproductive technologies (ART) come with uncertainty and side-effects, variable success rates, and are often prohibitively costly, amplifying the burden of infertility and limiting access to care (3).

While infertility can result from various biological causes, modifiable lifestyle factors - including suboptimal diet, physical inactivity, substance use, obesity and stress - are increasingly recognised as contributors to infertility and ART outcomes (4, 5). Poor preconception diets, in particular, have been linked to unfavourable fertility outcomes, with inflammation emerging as a key putative mechanism associated with infertility (6–8, 9). Diets high in pro-inflammatory components, such as processed meats, ultra-processed foods, sugars, refined carbohydrates and saturated or trans fats, are associated with poorer fertility (10, 11), while anti-inflammatory and Mediterranean-style diets, rich in fruits, vegetables, healthy fats, nuts, and fish, may improve fertility outcomes (11, 12).

Despite growing evidence linking diet and fertility, large-scale observational data examining the inflammatory potential of diet in relation to fertility remain limited (11). Although Mediterranean and anti-inflammatory diets closely align with general population-level healthy eating guidelines (both promote balanced consumption of fruits, vegetables, whole grains, lean proteins and healthy fats, while limiting refined sugars, salt and saturated fats), the comparative relationships of these diets in the context of fertility is unclear (13). To date, no studies have directly compared these dietary measures and their relative associations with fertility outcomes within a large population-based cohort.

Addressing this gap requires a comprehensive assessment of dietary patterns using multiple methods. A priori indices, such as the Healthy Eating Index or the Mediterranean Diet score, reflect adherence to established guidelines or to traditional diets, respectively, which have been linked with health benefits (14). Other indices such as the dietary inflammatory index (DII^®^) (15) are based on biological function, and were recently updated to address methodological concerns, namely in relation to total energy and nutrient intake, with the development of the now widely-adopted energy-adjusted DII (E-DII) (16). Alternatively, a posteriori methods such as principal component analysis (PCA) are exploratory, data-driven techniques used to empirically derive dietary patterns (12). While most studies rely on a single approach, employing both a priori and a posteriori methods offers methodological advantages, integrating both hypothesis-driven and data-driven explorations (17–19). This integrative approach, not previously applied in the fertility context, can enable rigorous comparisons to potentially identify novel diet-disease associations.

In this study, we aimed to examine whether dietary intake, assessed by both a priori and a posteriori dietary patterns, is cross-sectionally associated with self-reported fertility in a large population-based female cohort and whether these associations vary by dietary pattern scoring method.

## 2 Methods

### 2.1 Study design and participants

The Australian Longitudinal Study on Women’s Health (ALSWH) is a large population-based prospective cohort study, which follows three different age groups of Australian female participants over time. Participants were randomly selected from Medicare, Australia’s universal health insurance scheme which covers nearly all permanent residents of Australia, with some national recruitment and intentional over-sampling from rural and remote communities (20). The University of Newcastle and University of Queensland Human Research Ethics Committees approved the study protocol, and all participants provided written informed consent.

**Figure 1:**
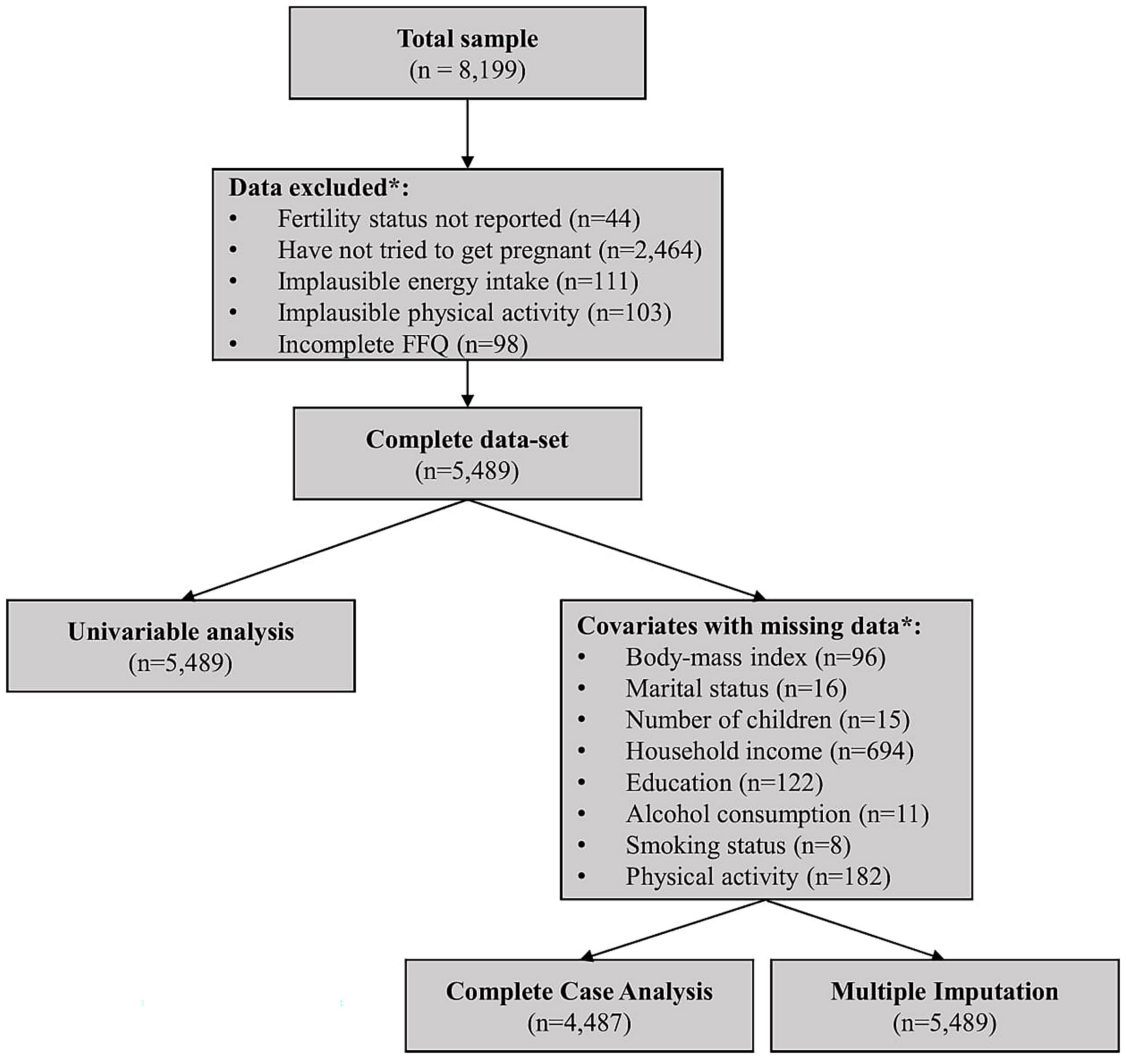
Flowchart depicting data exclusions and final sample sizes for univariable and multivariable analyses. *These categories overlap, with some women counted in multiple groups; implausible energy intake refers to <2,100 or >14,700 kJ/day (approximately <500 or >3,500 kcal/day), implausible physical activity refers to >1,680 min/week or >4 h/day; incomplete FFQ refers to >10% missing data. FFQ, food frequency questionnaire.

The ALSWH currently comprises nine surveys conducted from 1996 to the present day, involving participants born between 1921 and 1978. For the present study, we conducted cross-sectional analysis using data from ‘Survey 5’ (recruited in 1996 when participants were aged 18–23 years old), representing 8,199 female participants aged 31–36 years as of 2009. This survey and reporting period were selected because they provided the most complete measures of dietary intake compared to other surveys. We excluded participant data based on several criteria, with some individuals meeting more than one, resulting in overlapping exclusions (Figure 1). Specifically, we excluded those without the fertility outcome of interest (missing data *n* = 44 or have never tried to get pregnant, *n* = 2,464) and those with an incomplete dietary questionnaire (>16 items or 10% missing, *n* = 98). Participants with reported daily energy intakes or physical activity levels outside acceptable limits were also excluded (>14,700 kJ/day or <2,100 kJ/day of energy intake, *n* = 111; and >1,680 min/week or 4 h/day of physical activity, *n* = 103). These thresholds were informed by ALSWH recommendations (17–20) and are intended to minimise over- and under-reporting or data errors based on physiologically implausible values for sustained daily intake/ physical activity in adult women (15, 16). Following this, a total of 5,489 participants remained and were included in the analysis (Figure 1).

### 2.2 Exposure: dietary patterns

Participants provided information about their dietary intake using the Dietary Questionnaire for Epidemiological Studies (DQES) Version 2, a food frequency questionnaire (FFQ) developed by The Cancer Council of Victoria (21) and previously validated for use in young Australian female populations (22). Validation studies have shown that the DQES v2.0 provides reasonable estimates of food group consumption at the population level (23), with over 60% of participants classified into the same or adjacent quintiles for most food groups when compared to weighed food records in a young Australian population (24). Using a ten-point scale ranging from ‘never’ to ‘three or more times daily’, participants provided details on how often they consumed 74 food items and six types of alcoholic beverages over the previous 12 months. For vegetables, meats, and casseroles, serving sizes were reported using photographs depicting different portion sizes. Questions regarding the quantity and types of milk, bread, sugar, eggs, fat spreads, and cheese were also included, adding 21 more items to the FFQ. Responses to the FFQ were used to calculate daily consumption of a total of 101 food items (g/day) and their nutrient content, using data from NUTTAB95, a comprehensive database of Australian food compositions (25). These 101 food items were then categorised into 34 food groups, following a classification system used previously in an Australian cohort (26), to facilitate the analysis of dietary patterns.

#### 2.2.1 Energy-adjusted DII

The DII is an a priori diet quality index created to assess the inflammatory potential of a diet (15). Since its introduction, the DII has been validated using a variety of inflammatory biomarkers in over 50 studies, showing that higher DII scores were significantly associated with higher levels of circulating pro-inflammatory cytokines (27–30). Per the methodology described by Shivappa et al. (15), DII scores are based on an extensive database developed with rigorous literature review and analysis of 11 international datasets, with weighting dependent on the quality of the study designs from which this result was derived (15). However, as the DII can be influenced by variations in energy intake, the E-DII was developed to account for this confounding and normalise values relative to energy intake. We chose to use the E-DII as opposed to empirically derived indices (30) because the E-DII is one of the most widely used, adaptable, and extensively validated indices for assessing dietary inflammatory potential across diverse populations and disease outcomes, including reproductive health (27, 31–34). The E-DII is calculated using a density-based approach, where DII values are standardized per 1,000 kcal of dietary intake. This approach follows the same scoring methodology as the unadjusted DII, but relies on an energy-adjusted reference database (16). Using this method and the self-report values for 25 of the 45 DII food parameters available from the FFQ, the E-DII scores were calculated for all participants, and ranged from approximately −9 (a minimally inflammatory dietary pattern) to +8 (a maximally pro inflammatory diet).

#### 2.2.2 Dietary guideline index

The DGI is a food-based diet quality index that quantifies adherence to the Dietary Guidelines for Australian Adults, with higher scores indicating greater adherence with these guidelines (35). The DGI was calculated using the same method described in McNaughton et al. (36). Briefly, the DGI consists of 15 dietary indicators to characterise consumption of a variety of foods including fruits, vegetables, cereals, whole-grain cereals, meat and meat alternatives, lean protein sources, dairy foods, low-fat or reduced-fat dairy, fluids, as well as indicators for saturated fat intake, salt use, alcoholic beverages, added sugars and extra foods (defined as non-essential items that do not contribute significantly to meeting nutrient requirements, such as sugar-sweetened beverages, confectionery, discretionary foods, and others) (36). Each dietary indicator is given a score from 0 to 10, with a total cumulative score ranging from 0 to 150 (higher scores indicating increasing compliance with healthy eating guidelines) (36). Since the FFQ did not obtain specific information regarding salt use, fluid intake, and saturated fat components (trimming of fat from meat), these aspects were subsequently excluded (36).

#### 2.2.3 Principal component analysis

PCA was used to derive *a posteriori* dietary patterns, utilising factor loadings extracted from the principal component method and varimax/orthogonal rotation. A total of 34 food groups were included in the PCA. The number of dietary patterns was identified based on factors with eigenvalues >1.5, a break in the scree plot, and the interpretability and meaningfulness of the patterns (Supplementary Figure S1) (37, 38). Accordingly, a three-factor solution was selected, with food items displaying factor loadings >0.20 deemed relevant to the specific factor, and representing the foods most strongly associated with the pattern (39). Patterns were described in relation to the food groups that loaded most positively or negatively for those respective patterns (37). This method has been previously validated to assess diet quality in states of inflammation (38).

For the purpose of assessing whether the correlations were appropriate for PCA, we conducted the Kaiser-Meyer-Olkin sampling adequacy test and Bartlett’s test for Sphericity. This test aims to quantify the degree of intercorrelations among the variables, with a value >0.6 considered suitable for PCA (40). The Bartlett’s test for Sphericity aims to determine whether the variables are unrelated, with *p* < 0.05 indicating that there are significant correlations among at least some of the variables, justifying the use of PCA (40).

### 2.3 Outcome: fertility

Fertility problems were determined based on responses to the question: “Have you and your partner (current or previous) ever had problems with fertility – that is, tried unsuccessfully for 12 months or more to get pregnant?.” Response options included: ‘No, have never tried to get pregnant’; ‘No, have had no problem with fertility’; ‘Yes and have sought help/ treatment’; or ‘Yes, but have not sought help/ treatment’. This outcome, denoted herein using the term ‘fertility problems’, captures self-reported lifetime prevalence of infertility (i.e., difficulty conceiving), rather than incident infertility or current in/fertility status, and does not equate to a clinical diagnosis of infertility. As noted above, those who indicated they had never tried to get pregnant were excluded from the analysis. Data were categorised per previous ALSWH studies assessing this variable (41), whereby those who reported fertility problems (regardless of whether or not they sought assistance) were classified as the ‘fertility problems’ group, while those reporting no problems with fertility were classified as the ‘no fertility problems’ group.

### 2.4 Confounding variables

Sociodemographic and lifestyle-related variables were collected across different surveys and those relevant to our analysis were identified *a priori* using directed acyclic graphs (DAGs) on the basis of evidence and/or biological plausibility. These included maternal age (years); body-mass index (BMI, kg/m^2^); number of children (categorised as: ‘no children’, ‘1 child’, ‘2–3 children’ and ‘>3 children’); household income (categorised as: ‘low’, ‘medium’ and ‘high income’); education (categorised as: ‘no formal qualification or year 10/12 equivalent’, ‘trade/ apprenticeship’, ‘certificate/diploma’ and ‘degree or higher’); marital status (categorised as: ‘married/de facto’, ‘separated/divorced’, ‘widowed’ and ‘never married’); smoking status (categorised as: ‘never smoker’, ‘ex-smoker’ and ‘current smoker’); alcohol consumption (categorised as: ‘never drinks’, ‘<once a month’, ‘<once a week’, ‘1 or 2 days/week’, ‘3, 4, 5 or 6 days/week’ and ‘every day’); and physical activity (categorised as: ‘nil/sedentary <33.3 met-min/week’, ‘low 33.3 ≤ x < 500 met-min/week’, ‘medium 500 ≤ x < 1,000 met-min/week’ and ‘high 1,000 ≤ met-min/week’). Participants were also asked whether they had been diagnosed or treated for a range of conditions in the last three years. Based on responses to this question, two conditions, polycystic ovary syndrome (PCOS) and endometriosis, which are relevant to fecundability and, ultimately, fertility, were categorised (yes/no) and included as possible confounders in exploratory analyses.

### 2.5 Statistical analysis

STATA SE 18 (College Station, TX: StataCorp LLC.) was used for the statistical analysis. Frequencies and descriptive statistics were denoted by counts (and percentages) or means ± standard deviations (SD). Normality of distributions for all independent continuous variables were assessed by visual inspection of histograms, as well as inspection of the skewness and kurtosis values, which is a reliable method for large sample sizes (*n* > 300) (42). To compare sample characteristics between those with and without fertility problems, χ^2^ tests were performed for categorical variables and independent samples t-tests or Mann–Whitney U tests were used for continuous data, depending on whether data were normally or non-normally distributed, respectively.

To characterise differences in diets across multiple groups (i.e., quartiles) for the E-DII and DGI, analysis of variance or Kruskal-Wallis with post-hoc Dunn’s test were used for continuous data, depending on whether the data were normally or non-normally distributed, respectively, whereas χ^2^ tests were performed for categorical variables. Simple and multivariable logistic regression analyses were used to examine the relationship between fertility problems (yes/no) and the independent variables corresponding to each E-DII, DGI, or dietary pattern principal component score, all modelled as both continuous data (per-1 unit increase in diet score) and in quartiles, with adjustment for relevant confounding variables (covariates). Associations were expressed as odds ratios (OR) or adjusted ORs (aOR) with 95% confidence intervals (CI). For continuous data, estimates reflect the OR per one-unit increase in the predictor (dietary pattern). For quartile-based models, ORs were calculated relative to the lowest quartile (Q1), which served as the reference category. Effect sizes and corresponding 95% CIs were also used to compare the relative association between each dietary pattern/score and female fertility. Covariates were determined a priori based on the DAGs and biological plausibility, as described above. The primary multivariable model included maternal age, BMI, number of children, marital status, household income, alcohol consumption, smoking and physical activity. To ensure the reliability of the model and to reduce the likelihood of multicollinearity, the variance inflation factor was assessed (where <5 was deemed as low multicollinearity). As a sensitivity analysis, separate models were examined excluding BMI or alcohol consumption as covariates, given the potential role of BMI as a mediator in the relationship between diet and fertility and the contribution of alcohol as a component of the dietary measures assessed. Additional sensitivity analyses were also conducted using the primary model with adjustment for PCOS or endometriosis status.

Missing covariate data in the primary model were addressed in sensitivity analyses with multiple imputation using chained equations (MICE). A multivariate imputation model included all variables used in the analysis, with continuous variables (e.g., BMI) imputed using linear regression and categorical variables (e.g., alcohol consumption, income, education, marital status, smoking status, and physical activity) imputed using multinomial logistic regression. Principal components derived from dietary intake, the E-DII, and the DGI were included as predictors.

## 3 Results

### 3.1 Participant characteristics

Participant characteristics are reported in Table 1. After exclusion of missing and irrelevant data, 5,489 participants were included in the analysis (1,289 with fertility problems and 4,200 without fertility problems based on self-report). Participants with fertility problems had a higher body weight, BMI and waist circumference, fewer children, and were more likely to have PCOS or endometriosis, compared to those without fertility problems. There was no difference in physical activity level, total energy intake, frequency of alcohol consumption, smoking status, household income, highest qualification completed, or marital status between those with and without fertility problems.

**Table 1:** Sample characteristics stratified by self-reported fertility problems.

Characteristic	Fertility problems (*n* = 1,289)	No fertility problems (*n* = 4,200)
Age (years)	33.79 ± 1.44	33.83 ± 1.45
Anthropometric		
Weight (kg)	69.0 (60.0–82.0)	67.0 (60.0–78.0)
BMI (kg/m^2^)	24.95 (22.0–29.70)	24.40 (21.70–28.30)
Waist circumference (cm)	88.25 ± 14.65	86.50 ± 13.64
Physical activity (metabolic minutes/week)		
Overall	399.60 (116.50–999.0)	449.55 (149.85–999.0)
Nil/sedentary (<33.3)	190 (15.31)	584 (14.36)
Low (33.3 ≤ x < 500)	516 (41.58)	1,660 (40.83)
Medium (500 ≤ x < 1,000)	266 (21.43)	916 (22.53)
High (1000≤)	269 (21.68)	906 (22.28)
Total energy intake (kJ/day)	6,919.83 ± 2275.68	6,996.22 ± 2272.98
Frequency of alcohol consumption, *n* (%)		
Never drinks	183 (14.21)	565 (13.48)
< Once a month	361 (28.03)	1,057 (25.23)
< Once a week	284 (22.05)	935 (22.32)
1 or 2 days/week	243 (18.87)	830 (19.81)
3–6 days/week	188 (14.60)	712 (16.99)
Every day	29 (2.25)	91 (2.17)
Smoking status, *n* (%)		
Never smoker	771 (59.95)	2,499 (59.57)
Ex-smoker	358 (27.84)	1,162 (59.57)
Current smoker	157 (12.21)	534 (12.73)
Household income, *n* (%)		
Low income (AUD $1–36,999)	77 (6.74)	277 (7.58)
Medium income (AUD $37,000- 77,999)	353 (30.88)	1,081 (29.60)
High income (AUD > $77,999)	713 (62.38)	2,294 (62.81)
Highest qualification completed, *n* (%)		
No formal qualification or year 10/12 equiv.	306 (24.13)	954 (23.27)
Trade/ apprenticeship	33 (2.60)	122 (2.98)
Certificate/ diploma	337 (26.58)	979 (23.88)
Degree or higher	592 (46.69)	2,044 (49.87)
Marital status, *n* (%)		
Married/de Facto	1,188 (92.45)	3,826 (91.36)
Separated/divorced	60 (4.67)	206 (4.92)
Widowed	5 (0.39)	6 (0.14)
Never married	32 (2.49)	150 (3.58)
Number of children, *n* (%)		
No children	378 (29.37)	315 (7.52)
1	354 (27.51)	1,088 (25.99)
2–3	517 (40.17)	2,561 (61.17)
>3	38 (2.95)	223 (5.33)
PCOS, *n* (%)		
PCOS	327 (25.43)	209 (5.00)
No PCOS	959 (74.57)	3,969 (95.00)
Endometriosis, *n* (%)		
Endometriosis	147 (12.26)	84 (2.19)
No Endometriosis	1,052 (87.74)	3,758 (97.81)

Continuous data are expressed as mean ± standard deviation or median (interquartile range) and categorical data are expressed as frequencies (*n*, %). Differences between women with and without fertility problems were analysed with Mann Whitney-U or independent samples t-tests depending on normality for continuous variables and a χ^2^ test for categorical variables. ALSWH data reflects responses in Survey 5 (1973–1978); numbers may vary due to missing baseline values. BMI, body-mass index; kJ, kilojoule; PCOS, polycystic ovary syndrome.

### 3.2 Dietary intake

Characteristics of study participants according to E-DII, DGI, and PCA quartiles are presented in Supplementary Tables S1–S3, respectively, with associations in Tables 2–4. For E-DII scores, the mean ± SD for the cohort was −0.25 ± 1.37, indicating a marginally anti-inflammatory diet when adjusting for energy. Quartiles for E-DII were defined as: Q1 (−3.29 to −0.98), Q2 (−0.98 to −0.06), Q3 (−0.06 to 0.91) and Q4 (0.91 to 4.25), where Q1 and Q4 represent the lowest and highest dietary inflammatory potential after adjusting for energy, respectively. The mean DGI score for the cohort was 86.97 ± 11.27. Quartiles for DGI were defined as: Q1 (38.37 to 79.44), Q2 (79.45 to 87.70), Q3 (87.70 to 95.17) and Q4 (95.18 to 122.15), where Q1 and Q4 represent the lowest and highest adherence to healthy eating guidelines, respectively.

**Table 2:** Univariable and multivariable (adjusted) odds ratios for associations between energy-adjusted dietary inflammatory index, and dietary guideline index with self-reported fertility problems.

Diet index scores	Univariable			Multivariable (Adjusted)^2^		
	*n*	OR	95% CI	n	aOR	95% CI
E-DII Overall^1^	5,489	1.05	1.01, 1.10	4,487	1.13	1.06, 1.19
E-DII Quartiles						
Q1 (−4.41 – −0.98)	1,373	Reference		1,132	Reference	
Q2 (−0.98 – −0.06)	1,372	1.08	0.90, 1.29	1,140	1.10	0.89, 1.36
Q3 (−0.06–0.91)	1,372	1.14	0.96, 1.37	1,145	1.40	1.14, 1.73
Q4 (0.91–4.25)	1,372	1.23	1.04, 1.48	1,070	1.53	1.23, 1.90
sDGI Overall ^1^	5,489	1.00	0.99, 1.00	4,487	0.99	0.99, 0.99
DGI Quartiles						
Q1 (38.37–79.44)	1,373	Reference		1,053	Reference	
Q2 (79.45–87.70)	1,372	1.04	0.87, 1.24	1,116	0.96	0.78, 1.18
Q3 (87.70–95.17)	1,372	1.02	0.86. 1.22	1,155	0.96	0.78, 1.19
Q4 (95.18–122.15)	1,372	0.88	0.74, 1.06	1,163	0.76	0.61, 0.95

Logistic regression was conducted to derive the odds ratios for associations between dietary inflammatory index or dietary guideline index (overall and quartiles) with self-reported fertility problems. Differences in sample sizes between the crude and adjusted models are due to missing covariate data. Q1 was designated as the reference category. ^1^ reflects associations with overall (continuous) dietary pattern, with ORs representing a per-1 unit increase in the listed pattern. ^2^The model was adjusted for maternal age, body mass index, marital status, number of children, household income, education, alcohol consumption, smoking status and physical activity. aOR, adjusted odds ratio; CI, confidence interval; DGI, dietary guideline index; E-DII, energy-adjusted dietary inflammatory index; OR, odds ratio; Q, quartile.

**Table 3:** Rotated factor loadings for each of the identified principal components for women and without fertility problems.

Variable	Western-style dietary pattern	Mediterranean-style dietary pattern	Plant-dominant dietary pattern
Take away foods	**0.3431**	−0.0233	−0.1065
Cakes, biscuits, and sweet pastries	**0.3301**	−0.0464	0.0790
Processed meat	**0.3192**	−0.0556	0.0329
Crisps	**0.2970**	−0.0359	−0.0721
Refined grains	**0.2786**	0.0897	−0.0169
Red meat	**0.2583**	0.0242	0.1871
Fried fish	**0.2466**	0.0395	−0.0796
Poultry	**0.2388**	0.0460	0.0915
Confectionary	**0.2232**	0.0521	−0.0581
Vegemite	**0.2080**	0.0492	0.0410
All fish	0.1087	**0.2619**	−0.0795
Processed fish	0.1004	**0.2983**	−0.0993
Potatoes	0.0830	−0.1406	**0.4577**
Eggs	0.0671	**0.2006**	−0.0966
Nuts, nut spread	0.0607	**0.3227**	−0.0627
Wholegrains	0.0180	**0.2137**	0.0627
Yellow or green vegetables	0.0109	0.0810	**0.5107**
Other vegetables^1^	−0.0220	**0.3985**	0.1256
Legumes	−0.0332	0.0958	**0.3974**
Fresh fruit	−0.0431	**0.3259**	0.0412
Tomato	−0.0446	**0.2067**	0.0008
Garlic	−0.0494	**0.2689**	−0.0450
Leafy green vegetables	−0.0852	**0.3628**	0.0537
Cruciferous vegetables	−0.0975	0.0025	**0.4337**
Proportion	11.10%	8.07%	5.29%
Cumulative	24.46%		

Dietary patterns were identified using principal component analysis (PCA), a data reduction technique that groups correlated food items into underlying dietary patterns. PCA was conducted on dietary intake data from participants with complete responses (*n* = 5,489). Orthogonal (varimax) rotation was applied to improve interpretability by maximizing the variance explained by each factor while ensuring factors remained uncorrelated. Factor loadings represent the correlation between each food group and the derived dietary pattern. Factor loadings >0.20 are bolded. Food groups with loadings <0.20 for all factors are not listed (canned fruit, low fat dairy, full fat dairy, soya, added sugar, juice, tomato sauce, unsaturated spreads, saturated spreads and alcohol). ^1^Other vegetables category contains beetroot, celery, mushroom, onion, cucumber, and bean sprouts.

**Table 4:** Univariable and multivariable (adjusted) odds ratios for associations between principal component analysis-derived dietary pattern scores and self-reported fertility problems.

Principal component	Univariable			Multivariable (adjusted)^2^		
	*n*	OR	95% CI	*n*	aOR	95% CI
Western-style dietary pattern^1^	5,489	0.99	0.96, 1.03	4,487	1.02	0.98, 1.07
Quartiles						
Q1 (−4.48 to −1.28)	1,373	Reference		1,123	Reference	
Q2 (−1.28 to −0.28)	1,372	1.07	0.90, 1.27	1,145	1.13	0.92, 1.39
Q3 (−0.28 to 0.96)	1,372	0.97	0.81, 1.16	1,126	1.05	0.85, 1.29
Q4 (0.96 to 10.67)	1,372	1.03	0.86, 1.23	1,093	1.17	0.95, 1.45
Mediterranean-style dietary pattern^1^	5,489	0.95	0.92, 0.99	4,487	0.92	0.88, 0.97
Quartiles						
Q1 (−4.41 to −1.18)	1,373	Reference		1,095	Reference	
Q2 (−1.18 to −0.25)	1,372	0.98	0.82, 1.16	1,123	0.93	0.76, 1.14
Q3 (−0.24 to 0.92)	1,372	0.88	0.74, 1.05	1,129	0.83	0.67, 1.02
Q4 (0.92 to 10.53)	1,372	0.81	0.67, 0.96	1,140	0.69	0.56, 0.86
Plant-dominant dietary pattern^1^	5,489	0.96	0.92, 0.99	4,487	0.96	0.91, 1.01
Quartiles						
Q1 (−4.48 to −1.04)	1,373	Reference		1,088	Reference	
Q2 (−1.04 to −0.23)	1,372	0.90	0.76, 1.08	1,135	0.88	0.72, 1.08
Q3 (−0.23 to 0.81)	1,372	0.88	0.74, 1.04	1,149	0.91	0.74, 1.12
Q4 (0.81 to 12.50)	1,372	0.77	0.65, 0.92	1,115	0.77	0.63, 0.96

Logistic regression was conducted to derive the odds ratios between principal component analysis-derived dietary pattern scores (overall and quartiles) and self-reported fertility problems. ^1^reflects associations with overall (continuous) dietary pattern, with ORs representing a per-1 unit increase in the listed pattern. ^2^The model was adjusted for maternal age, body mass index, marital status, number of children, household income, education, alcohol consumption, smoking status, and physical activity. aOR, adjusted odds ratio; CI, confidence interval; OR, odds ratio.

Nutrient and whole food intakes by E-DII and DGI quartiles are presented in Supplementary Tables S4, S5, respectively. For E-DII, the highest quartile had the highest proportions of energy derived from total fat, saturated fat, and sugars, while having the lowest proportions from carbohydrates and protein. Those in Q4 also had higher dietary intakes of cholesterol, retinol, sodium, red and processed meats, poultry, full-fat dairy, refined grains, fried and takeaway foods, cakes, biscuits, sweet pastries, and confectionary. Conversely, participants in the lowest quartile had higher intakes of fibre, magnesium, potassium, iron, zinc, vitamins C and E, wholegrains, nuts and nut spreads, legumes, fresh fruit, and a variety of vegetables. For DGI, the lowest quartile (Q1, least healthy) had the highest proportions of energy (%) derived from fats [except polyunsaturated fatty acids (PUFAs)] and sugars, with the highest dietary intakes of cholesterol, retinol, refined grains, red and processed meats, poultry, potatoes, eggs, full fat dairy, alcohol, confectionary, cakes, biscuits and sweet pastries and takeaway foods.

PCA revealed three distinct patterns accounting for 24.46% of total variance (Table 3). Component 1 is labelled as a “Western style” dietary pattern due to the high loadings of red and processed meat, take away foods, and ultra-processed foods. Component 2 is labelled as a “Mediterranean style” dietary pattern due to food group loadings including various vegetables, fresh fruit, nuts, whole grains, and fish. Component 3 is labelled as a “plant-dominant” dietary pattern due to high loading of potato, yellow or green vegetables, legumes and cruciferous vegetables. The quartiles for the PCA-derived dietary pattern scores were defined as follows: for the Western-style dietary pattern: Q1 (−4.48 to −1.28), Q2 (−1.28 to −0.28), Q3 (−0.28 to 0.96), and Q4 (0.96 to 10.67); for the Mediterranean-style dietary pattern: Q1 (−4.41 to −1.18), Q2 (−1.18 to −0.25), Q3 (−0.24 to 0.92), and Q4 (0.92 to 10.53); and for the plant-dominant dietary pattern: Q1 (−4.48 to −1.04), Q2 (−1.04 to −0.23), Q3 (−0.23 to 0.81), and Q4 (0.81 to 12.50). These scores indicate how closely an individual’s dietary intake aligns with each respective dietary pattern, with higher scores reflecting greater adherence.

### 3.3 Associations between dietary measures and demographic variables

Those in the highest quartile of the E-DII (Q4, most inflammatory) had higher body weight, BMI, and waist circumference compared to the lowest quartile (Q1, least inflammatory). Participants with higher E-DII scores were more likely to be current smokers and consume alcohol more frequently, whereas ‘never smoking’ and abstention/less frequent alcohol consumption was more prevalent among those in the lower quartiles. For socioeconomic variables, Q4 participants more likely to have lower household incomes, lower levels of formal education, a greater proportion of married or de facto individuals and more children, compared with Q1. No significant differences in age, PCOS status, or endometriosis were observed across E-DII quartiles (Supplementary Table S1).

For DGI scores, those in the highest (Q4, most healthy) quartiles had lower BMI and waist circumference and higher total energy intake and physical activity compared with the lowest DGI quartile (Q1, least healthy). The highest DGI quartile also had fewer current smokers, less frequent alcohol consumption, higher household income, and were more likely to be married, formally educated, and have no children or one child compared to the lowest DGI quartile. No significant differences in age, PCOS, or endometriosis were observed across DGI quartiles (Supplementary Table S2).

For PCA-derived patterns, those with the highest intake of the Western-style dietary pattern had higher weight, BMI, waist circumference, and energy intake, and more likely to be sedentary, current smokers and have no formal qualification, low income and more children. Conversely, those with higher intake of a Mediterranean-style dietary pattern had lower BMI and waist circumference, higher physical activity and energy intake, fewer children, less smoking, and were more likely to have a higher income and hold a degree. For the plant-dominant dietary pattern, the higher intake quartile had higher weight, BMI, waist circumference, energy intake and more children, but were less likely to frequently consume alcohol and more likely to be in the medium income and education brackets (Supplementary Tables S3A–C).

### 3.4 Associations between dietary measures and fertility

Results from regression analysis of the E-DII and the DGI with self-reported fertility problems are presented in Table 2 and Supplementary Table S6. Higher E-DII overall was associated with higher odds of self-reported fertility problems (OR per 1-unit increase in E-DII: 1.05; 95% CI: 1.01, 1.10, *p* = 0.045), including after adjustment for covariates in the primary model (aOR per 1-unit increase in E-DII: 1.13; 95% CI: 1.06, 1.19, *p* < 0.0001). When comparing quartiles of the E-DII, the odds of fertility problems were 23% higher for those in the most inflammatory quartile (Q4) compared with the least inflammatory (Q1) in univariable analysis (OR: 1.23; 95% CI: 1.04, 1.48, *p* = 0.02). With multivariable adjustment, there were 40 and 53% higher odds of fertility problems in Q3 and Q4 of the E-DII compared to Q1, respectively (aOR: 1.40; 95% CI: 1.14, 1.73, *p* = 0.002 and aOR: 1.53; 95% CI: 1.23, 1.90, *p* < 0.0001; Table 2). Results were not materially altered by excluding BMI or alcohol consumption from the primary model or by additionally adjusting for PCOS or endometriosis status (Supplementary Table S6).

For DGI, there was no association between overall DGI score and self-reported fertility problems in univariable analysis (Table 2), with a borderline association in adjusted analyses (aOR per 1-unit increase in DGI: 0.99; 95% CI: 0.99, 0.99, *p* = 0.047). Results were similar in sensitivity analyses excluding BMI or alcohol consumption from the model (Supplementary Table S6). When classified into quartiles, there were no differences in univariable analysis, but the odds of fertility problems were 24% lower in the highest DGI quartile (most healthy, Q4) compared with the lowest (least healthy, Q1) in adjusted analysis (aOR: 0.76; 95% CI: 0.61, 0.95, *p* = 0.02) (Table 2). Excluding BMI or alcohol consumption from the primary model or additionally adjusting for PCOS or endometriosis status did not alter these results (Supplementary Table S6).

As shown in Table 4, consumption of a Mediterranean-style dietary pattern was associated with 5% lower odds of fertility problems in univariable (OR per 1-unit increase in Mediterranean-style pattern: 0.95; 95% CI: 0.92, 0.99, *p* = 0.01), and 8% lower odds in multivariable analyses (aOR per 1-unit increase in Mediterranean-style dietary pattern: 0.92 95% CI: 0.88, 0.97, *p* = 0.001). When stratified into quartiles, the odds of fertility problems were 19 and 31% lower when comparing Q4 to Q1 in univariable and multivariable analyses (OR: 0.81; 95% CI: 0.67, 0.96, *p* = 0.02; and aOR: 0.69; 95% CI: 0.56, 0.86, *p* = 0.01, respectively). Consumption of a plant-dominant dietary pattern was also associated with lower self-reported fertility problems in univariable (OR per 1-unit increase in plant-dominant pattern: 0.96; 95% CI: 0.92, 0.99, *p* = 0.04), but not in adjusted, analysis (Table 4). When stratified into quartiles, the odds of fertility problems were 23% lower when comparing Q4 to Q1 in univariable and multivariable analyses (OR: 0.77; 95% CI: 0.65, 0.92, *p* = 0.01; and aOR: 0.77; 95% CI: 0.63, 0.96, p = 0.02, respectively). A Western-style dietary pattern was not associated with self-reported fertility problems in univariable or multivariable analyses, including when stratified by quartiles (Table 4). Sensitivity analyses excluding BMI or alcohol consumption from the primary model or additionally adjusting for PCOS or endometriosis status in the multivariable model (either overall or quartiles) did not alter the results for any of the PCA-derived dietary patterns (Supplementary Table S6).

In the sensitivity analysis using imputed data, the magnitude, direction, and significance of associations for the E-DII and DGI (overall and by quartiles) did not materially differ from the complete case analysis (Supplementary Table S7). For PCA-derived dietary patterns, higher consumption of a Western-style pattern (Q4 and Q2 vs. Q1) became significantly associated with higher odds of fertility problems, and Q3 of the Mediterranean-style pattern also became associated with lower odds of fertility problems compared with Q1 in the imputed analysis (Supplementary Table S7). No changes were observed for the plant-dominant dietary pattern with multiple imputation (Supplementary Table S7).

## 4 Discussion

To our knowledge, this is the first study to examine different dietary measures, including E-DII, DGI, and PCA-derived patterns, in relation to fertility problems in a large, population-based cohort. We report lower odds of fertility problems with consumption of a healthy diet, whether reflected by lower dietary inflammatory potential, adherence to a Mediterranean-style dietary pattern, or alignment with general dietary guidelines. These novel findings highlight the value of general healthy eating as a flexible alternative to more prescriptive dietary approaches for supporting female fertility.

Our data align with prior observational data from the US National Health and Nutrition Examination Survey (*n* = 2,066–2,613) and the Ravansar Non-Communicable Diseases study in Iran (*n* = 4,437), whereby higher DII (43–46) and E-DII (45) scores, indicating a more inflammatory diet, were independently associated with fertility problems. In our study, higher E-DII scores reflected higher intakes of sodium, cholesterol, total and saturated fats, refined grains, sugars, poultry, red and processed meat and take-away foods. Some of these individual components are linked with inflammation (47–49), and higher E-DII scores have been correlated with several inflammatory markers, including interleukin-6 and C-reactive protein (50, 51). In turn, elevated inflammation can promote insulin resistance and hormonal imbalances, and impair key reproductive functions including ovulation and endometrial receptivity (52–55). It is therefore plausible that high E-DII scores, reflecting diets with greater inflammatory potential, correlate with fertility problems, since optimal reproductive function depends on a balanced inflammatory response. However, as we did not directly measure inflammatory markers, this mechanism remains speculative and warrants further study.

Our data also showed that higher DGI scores, reflecting adherence to the Australian Guidelines for Healthy Eating (AGHE) in this study, were independently associated with reduced fertility problems. As a national evidence-based guideline, the AGHE recommends a diet rich in fruits, vegetables, legumes, whole grains, lean meats, fish, eggs, tofu, nuts and seeds, while limiting alcohol, ultra-processed foods and sugary drinks (13). In line with these recommendations, and also with the broad composition of traditional Mediterranean diets, higher DGI adherence in our study was associated with higher intakes of fruits, vegetables, wholegrains, legumes, nuts/nut spreads, fish, soya, and key nutrients such as magnesium, calcium, iron, and protein, among others. Conversely, lower DGI scores were associated with higher intakes of fats, sugars, cholesterol, refined grains, red and processed meats, take-aways, confectionary, cakes, biscuits and sweets, eggs, full fat dairy and alcohol. These patterns align with those captured by the E-DII and Mediterranean-style pattern, highlighting that potential benefits for fertility may stem from these specific food components, rather than any single dietary pattern.

Consistent with our E-DII and DGI findings, higher consumption of a Mediterranean-style dietary pattern was associated with lower odds of fertility problems. This aligns with prior observational data from IVF and general populations across the USA, China, Spain, Netherlands, and Greece (56–59). The Mediterranean-style dietary pattern, characterised here by high loadings of fresh fruit, vegetables, whole grains, nuts, and fish, is thought to improve reproductive health by reducing inflammation and oxidative stress and improving hormonal and metabolic regulation (60–63). Extra virgin olive oil is a key component of this diet, and is rich in oleocanthal, a compound structurally similar to the anti-inflammatory pharmacological agent ibuprofen (64). Another component is fish, which is rich in omega-3 fatty acids (e.g., polyunsaturated fatty acids or PUFAs) including docosahexaenoic acid (DHA) - known to promote anti-inflammatory and antioxidant effects (65), and thought to enhance ovulation (66, 67). A recent study reported that a higher intake of PUFAs, namely DHA and alpha-linolenic acid, was associated with improved fecundability and lower subfertility risk in females (68). Beyond inflammation, reduced intake of discretionary foods (e.g., processed meats and take-aways), may improve insulin sensitivity and hormonal balance (including regulating estrogen and progesterone and improving responses to follicle stimulating hormone) (69). Together, these endocrine functions can enhance fertility by promoting follicular development, ovulation and endometrial receptivity for implantation (11, 70–72). Indeed, consistent clinical evidence supports the benefits of traditional Mediterranean diets on female fertility (12), including live birth and clinical pregnancy, as shown in our prior systematic review (11). However, to our knowledge, only one study has compared healthy and anti-inflammatory diets using FFQ-based metrics (56), finding that the ‘fertility diet’, but not the Mediterranean diet, was associated with improved fertility outcomes among females undergoing ART. This population may differ in health behaviours and underlying pathologies, which could explain discrepancies with our findings (56). While fertility-specific diets were not assessed here, and thus cannot be directly compared, we extend prior evidence by demonstrating consistent associations between multiple dietary measures and fertility problems in a broader, community-based population, outside the ART context.

There is substantial conceptual overlap between anti-inflammatory diets, general healthy eating guidelines, and the Mediterranean diet (both traditional and PCA-derived patterns). All emphasise plant-based foods, grains, fish, nuts and seeds, while limiting red or processed meats, take-away foods and ultra-processed products. Given this overlap, and the similar associations found between these dietary measures and fertility problems, we posit that specific anti-inflammatory or Mediterranean-style diets may not offer unique benefits beyond those provided by broader guideline-recommended healthy diets. This contrasts with some meta-analyses (73, 74), which suggest the traditional Mediterranean diet confers distinct advantages for metabolic health and fertility, though such findings are often limited by heterogeneity and recall bias. Evidence for fertility-specific diets is also inconsistent, with some (56, 75, 76), but not all (56, 75) studies reporting fertility benefits from adherence to whole dietary patterns, such as the ‘fertility diet,’ ‘healthy dietary pattern’ and ‘pro-fertility diet’, which also largely align with Mediterranean diet principles (emphasising fruits, vegetables, wholegrains, legumes, etc.). While guideline-recommended diets may not include all the key features of more prescriptive Mediterranean diets, such as the focus on extra virgin olive oil, they may still provide comparable benefits by optimising core food and nutrient intake and limiting discretionary foods, as supported by the similar effect sizes observed across dietary patterns/scores in this study. Importantly, we did not assess fertility-specific dietary patterns such as the ‘fertility diet’ in this study due to limited FFQ data, and thus cannot draw conclusions about their relative effectiveness compared to the dietary indices used in this study.

This study has several strengths. To our knowledge, this is the first study to comprehensively assess several dietary measures, including E-DII, DGI, and PCA-derived patterns, in relation to fertility problems in a large population-based cohort, enhancing generalisability. All PCA assumptions were met (Supplementary Table S8), including sampling adequacy (77, 78) and an appropriate correlation matrix (77), supporting the validity of retained components despite low variance. Our robust analytical approach included detailed multivariable modelling, sensitivity analyses and MICE to address missing data - all with largely consistent results. We adjusted for key sociodemographic factors known to influence fertility, such as BMI (79), physical activity, smoking, alcohol intake (80, 81), marital status, income and education (82). However, other relevant factors such as genetics, stress and health conditions beyond PCOS or endometriosis, were not included, and may mediate or confound the observed relationships (4).

The main limitation of the study is its cross-sectional design, which precludes causal inference and may be affected by reverse causation. For instance, women experiencing fertility difficulties may have modified their diets in response to these challenges, and such changes could bias associations and complicate interpretation. Our exclusion of those who had never attempted pregnancy was necessary for outcome classification but may introduce selection bias. Additionally, our focus on females limits insights into male factors and their relative contributions or shared lifestyle behaviours that may influence fertility. Dietary data were collected at one time-point and, although diet is thought to be relatively stable in adulthood (83–85), this assumption cannot be verified in the current study. Similarly, our fertility assessment likely reflects lifetime prevalence of self-reported fertility problems, rather than incident infertility or current in/fertility status. This may capture past or present experiences, and does not distinguish between ongoing and resolved problems, warranting cautious interpretation regarding temporality of the observed relationships. Reliance on self-reported data for both fertility problems and dietary intake via FFQs may introduce recall or response bias and misclassification, although previous studies support the use of self-report as a generally reliable and practical method to assess infertility in epidemiological research (86–88). The FFQ used did not capture many components of the E-DII and DGI, including salt intake, sugar-sweetened beverages and saturated fats, which limits its application for a broader range of dietary patterns and may explain the lack of significant associations with the Western style dietary pattern in our study. Indeed, only 25 of the 45 E-DII/DII food parameters were available from the FFQ; however, this is within the range required for calculation (16, 89) and the computation was performed by the original developers (JRH). While consistent with previous studies, this approach may not fully capture all contributors to dietary inflammatory potential. Moreover, PCA is considered an exploratory rather than hypothesis-driven approach, and its processes - such as selecting food groups/items, determining the number of principal components, identifying which food groups have strong factor loadings, and, most importantly, classifying the components - are subjective in nature and require cautious interpretation (39). Lastly, as ORs from logistic regression are non-collapsible measures, differences in aORs across models (e.g., with or without BMI) may reflect statistical properties rather than confounding *per se*, and should be interpreted accordingly.

In summary, our novel findings suggest that healthy diets, characterised by either lower dietary inflammatory potential, adherence to a Mediterranean-style dietary pattern, or alignment with population-based dietary guidelines, are associated with lower odds of fertility problems. These approaches contain similar components, emphasising fruits, vegetables, wholegrains, legumes, nuts, seeds and fish, and limiting saturated fats, sugar, red and processed meats and take-away foods. These findings suggest that general, guideline-based healthy eating can support female fertility and may offer a flexible alternative to more prescriptive dietary approaches. Although effect sizes were modest, particularly for DGI, these were similar in magnitude to those observed in previous large-scale studies where comparable dietary improvements were associated with meaningful changes in clinical or biochemical markers (90–92). Thus, even small dietary improvements may yield clinically relevant benefits at a population level, given the high prevalence of fertility problems and the feasibility of dietary change. Future longitudinal studies incorporating repeated dietary assessments and biomarker validation are warranted to confirm temporality, strengthen causal inference, and provide mechanistic insights into the role of diet in female fertility.

## Data Availability

The data analyzed in this study is subject to the following licenses/restrictions: data described in the manuscript, code book, and analytic code is available upon request pending approval by the Australian Longitudinal Study on Women’s Health (ALSWH). Requests to access these datasets should be directed to alswh@newcastle.edu.au.
