# Supplementary material for: Diet and female fertility: a population-based study re-evaluating the need for prescriptive dietary patterns
Source: Front Nutr. 2025 Oct 22;12:1682549. doi: 10.3389/fnut.2025.1682549 (PMC12586016; doi:10.3389/fnut.2025.1682549)
Supplement: Supplementary file 1 [file Table_1.DOCX]

**Supplement to: Alesi et al. Diet and female infertility: a population-based study re-evaluating the need for prescriptive dietary patterns**

**
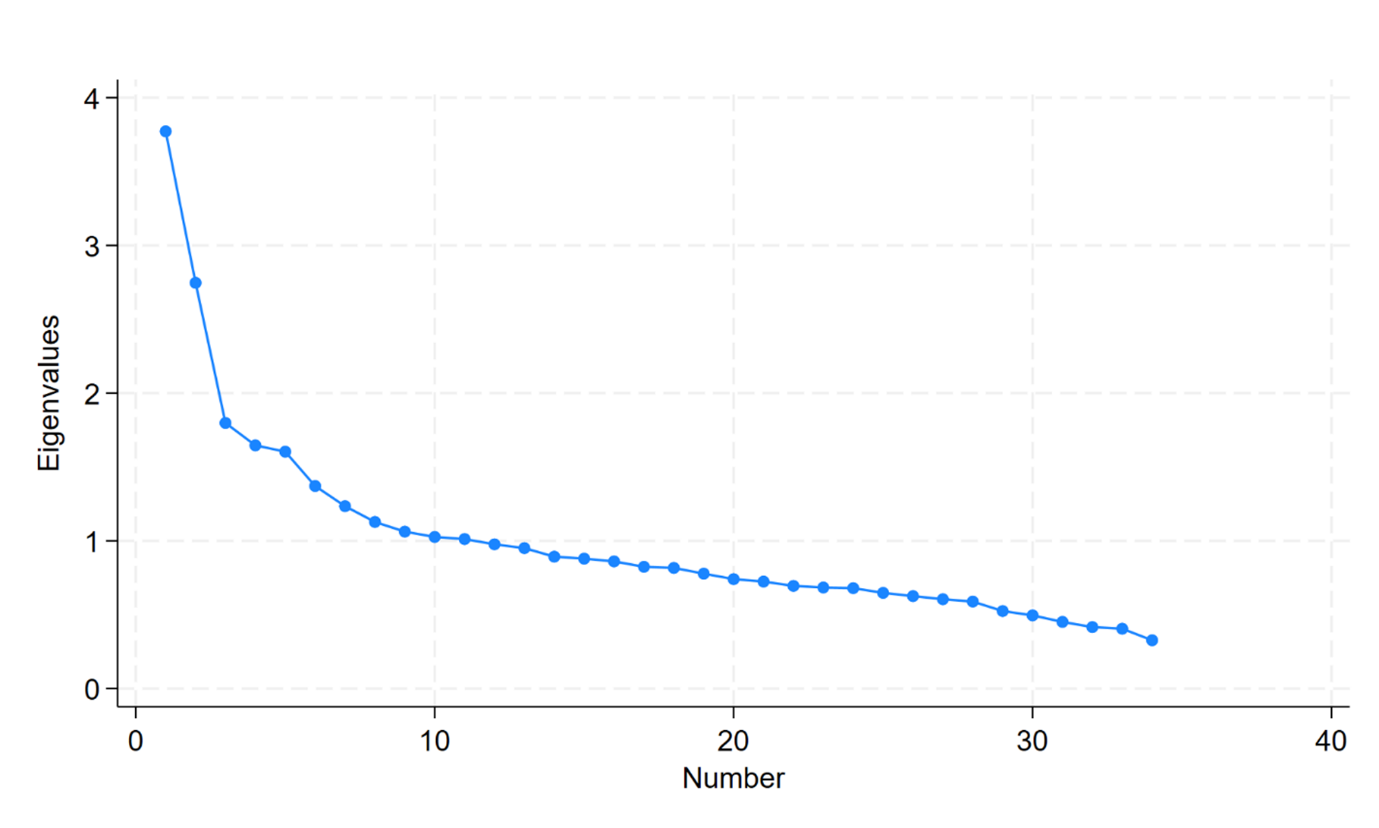
**

**Figure S1:** Scree plot of principal component analysis (PCA) showing the proportion of variance explained by each principal component. The x-axis represents the number of principal components in descending order, while the y-axis indicates the eigenvalue attributed to each principal component.

**Table S1**. Baseline characteristics of women at survey 5 (1973-1978) stratified by quartile of energy-adjusted dietary inflammatory index (E-DII).

| **Baseline characteristic** | **E-DII** | | | | ***p*** |
| --- | --- | --- | --- | --- | --- |
|  | **Q1**  **(-4.41 – -0.98)** | **Q2**  **(-0.98 – -0.06)** | **Q3**  **(-0.06 – 0.91)** | **Q4**  **(0.91 – 4.25)** |  |
| ***n*** | **1,373** | **1,372** | **1,372** | **1,372** |  |
| **Anthropometry** | | | | | |
| Age (years) | 33.86 ± 1.43 | 33.76 ± 1.45 | 33.85 ± 1.45 | 33.80 ± 1.46 | 0.26 |
| Weight (kg) | 67.0 (59.0-76.0) | 67.0 (60.0-80.0) | 68.0 (60.0-79.0) * | 70.0 (60.0-82.0) ** | 0.0001 |
| BMI (kg/m^2^) | 24.0 (21.50-27.60) | 24.30 (21.50-28.50) | 24.50 (22.0-28.50) ** | 25.30 (22.20-29.80) ** | 0.0001 |
| Waist circumference (cm) | 84.33 ± 12.68 | 86.69 ± 13.87 * | 87.33 ± 13.26 ** | 89.47 ± 15.31** | <0.0001 |
| **Physical activity (metabolic minutes/week)** | | | | | |
| Nil/sedentary (<33.3) | 131 (9.82) | 169 (12.75) | 176 (13.29) | 298 (22.51) | <0.0001 |
| Low (33.3 ≤ x < 500) | 469 (35.16) | 540 (40.75) | 598 (45.17) | 569 (42.98) |  |
| Medium (500 ≤ x < 1000) | 321 (24.06) | 315 (23.77) | 300 (22.66) | 246 (18.58) |  |
| High (1000≤) | 413 (30.96) | 301 (22.72) | 250 (18.88) | 211 (15.94) |  |
| **Total energy** | | | | | |
| Total energy intake (kJ/day) | 6066.82 ± 2022.03 | 6845.01 ± 2061.19 | 7223.22 ± 2188.29 | 7778.75 ± 2468.51 | <0.0001 |
| **Frequency of alcohol consumption** | | | | | |
| Never drinks | 147 (10.74) | 176 (12.85) | 195 (14.24) | 230 (16.79) | <0.0001 |
| < Once a month | 312 (22.79) | 359 (26.20) | 342 (24.98) | 405 (29.56) |  |
| < Once a week | 322 (23.52) | 284 (20.73) | 306 (22.35) | 307 (22.41) |  |
| 1 or 2 days/week | 302 (22.06) | 292 (21.31) | 259 (18.92) | 220 (16.06) |  |
| 3/4/5/6 days/week | 252 (18.41) | 233 (17.01) | 233 (17.02) | 182 13.28) |  |
| Every day | 34 (2.48) | 26 (1.90) | 34 (2.48) | 26 (1.90) |  |
| **Smoking classification** | | | | | |
| Never smoker | 829 (60.47) | 876 (63.99) | 803 (58.61) | 762 (55.58) | <0.0001 |
| Ex-smoker | 440 (32.09) | 347 (25.35) | 389 (28.39) | 344 (25.09) |  |
| Current smoker | 102 (7.44) | 146 (10.66) | 178 (12.99) | 265 (19.33) |  |
| **Household income** | | | | | |
| Low income ($AUD 1 – 36,999) | 75 (6.24) | 69 (5.70) | 87 (7.10) | 123 (10.63) | <0.0001 |
| Medium income ($AUD 37,000– 77,999) | 326 (27.12) | 350 (28.93) | 373 (30.42) | 385 (33.28) |  |
| High income ($AUD > 77,999) | 801 (66.64) | 791 (65.37) | 766 (62.48) | 649 (56.09) |  |
| **Highest qualification completed** | | | | | |
| No formal qualification or year 10/12 equiv. | 233 (17.48) | 300 (22.21) | 311 (23.19) | 416 (31.00) | <0.0001 |
| Trade/ apprenticeship | 31 (2.33) | 33 (2.44) | 47 (3.50) | 44 (3.28) |  |
| Certificate/ diploma | 308 (23.11) | 299 (22.13) | 327 (24.38) | 382 (28.46) |  |
| Degree or higher | 761 (57.09) | 719 (53.22) | 656 (48.92) | 500 (37.26) |  |
| **Marital status** | | | | | |
| Married/De Facto | 1,241 (90.58) | 1,265 (92.54) | 1,275 (93.13) | 1,233 (90.20) | 0.02 |
| Separated/ divorced | 82 (5.99) | 63 (4.61) | 56 (4.09) | 65 (4.75) |  |
| Widowed | 3 (0.22) | 3 (0.22) | 1 (0.07) | 4 (0.29) |  |
| Never married | 44 (3.21) | 36 (2.63) | 37 (2.70) | 65 (4.75) |  |
| **Number of children** | | | | | |
| No children | 243 (17.74) | 196 (14.31) | 137 (10.03) | 117 (8.55) | <0.0001 |
| 1 | 374 (27.30) | 358 (26.13) | 375 (27.45) | 335 (24.49) |  |
| 2-3 | 705 (51.46) | 763 (55.69) | 790 (57.83) | 820 (59.94) |  |
| >3 | 48 (3.50) | 53 (3.87) | 64 (4.69) | 96 (7.02) |  |
| **PCOS status** | | | | | |
| No PCOS | 1,221 (89.52) | 1,236 (90.62) | 1,246 (91.08) | 1,225 (89.55) | 0.42 |
| PCOS | 143 (10.48) | 128 (9.38) | 122 (8.92) | 143 (10.45) |  |
| **Endometriosis** | | | | | |
| No endometriosis | 1,190 (94.44) | 1,203 (95.32) | 1,200 (96.46) | 1,217 (95.45) | 0.12 |
| Endometriosis | 70 (5.56) | 59 (4.68) | 44 (3.54) | 58 (4.55) |  |

Continuous data are expressed as mean ± standard deviation or median (interquartile range), and categorical variables are expressed as frequency (within-column relative frequencies). Numbers may vary due to missing baseline values. Data were analysed with an analysis of variance (ANOVA) or Kruskal-Wallis with post-hoc Dunn’s test for multiple comparisons for normal or non-normal data, respectively. χ^2^ tests were used for categorical variables. P represents the result from the ANOVA, Kruskal-Wallis test, or χ^2^ test, with two-tailed p<0.05 considered statistically significant. *P<0.05 for Q2 vs Q1 and Q3 vs Q1 for waist circumference and BMI, respectively. **P<0.0001 for Q4/Q3 vs Q1 for BMI. **BMI,** body mass index**; IVF,** in-vitro fertilisation; **kJ,** kilojoule; **PCOS,** polycystic ovary syndrome; **Q,** quartile.

**Table S2**. Baseline characteristics of women at survey 5 (1973-1978) stratified by quartile of dietary guideline index (DGI) score.

| **Baseline characteristic** | **Dietary guideline index** | | | |  |
| --- | --- | --- | --- | --- | --- |
|  | **Q1**  **(38.37 – 79.44)** | **Q2**  **(79.45 - 87.70)** | **Q3**  **(87.70 - 95.17)** | **Q4**  **(95.18 - 122.15)** | ***p*** |
| **n** | **1,373** | **1,372** | **1,372** | **1,372** |  |
| **Anthropometry** | | | | | |
| Age (years) | 33.89 ± 1.44 | 33.82 ± 1.45 | 33.80 ± 1.46 | 33.76 ± 1.45 | 0.12 |
| Weight (kg) | 68.0 (60.0-80.0) | 68.0 (60.0-80.0) | 67.0 (60.0-78.0) | 66.0 (59.0-79.0) | 0.11 |
| BMI (kg/m^2^) | 25.0 (21.80-29.30) | 24.60 (22.0-28.60) | 24.20 (21.80-28.10) ** | 24.20 (21.60-28.30) ** | 0.009 |
| Waist circumference (cm) | 87.96 ± 14.91 | 86.95 ± 13.45 | 86.51 ± 13.64 | 86.28 ± 13.56* | 0.03 |
| **Physical activity (metabolic minutes/week)** | | | | | |
| Nil/sedentary (<33.3) | 298 (22.56) | 198 (14.93) | 165 (12.48) | 113 (8.45) | <0.0001 |
| Low (33.3 ≤ x < 500) | 545 (41.26) | 578 (43.59) | 509 (38.50) | 544 (40.66) |  |
| Medium (500 ≤ x < 1000) | 244 (18.47) | 288 (21.72) | 339 (25.64) | 311 (23.24) |  |
| High (1000≤) | 234 (17.71) | 262 (19.76) | 309 (23.37) | 370 (27.65) |  |
| **Total energy** | | | | | |
| Total energy intake (kJ/day) | 6660.74 ± 2450.75 | 6754.68 ±2246.36*** | 7000.06 ± 2225.11*** | 7497.88 ± 2064.76*** | <0.0001 |
| **Frequency of alcohol consumption** | | | | | |
| Never drinks | 179 (13.06) | 169 (12.35) | 189 (13.80) | 211 (15.41) | <0.0001 |
| < Once a month | 327 (23.85) | 341 (24.93) | 356 (25.99) | 394 (28.78) |  |
| < Once a week | 251 (18.31) | 290 (21.20) | 332 (24.23) | 346 (25.27) |  |
| 1 or 2 days/week | 253 (18.45) | 265 (19.37) | 262 (19.12) | 293 (21.40) |  |
| 3/4/5/6 days/week | 300 (21.88) | 270 (19.74) | 213 (15.55) | 117 (8.55) |  |
| Every day | 61 (4.45) | 33 (2.41) | 18 (1.31) | 8 (0.58) |  |
| **Smoking classification** | | | | | |
| Never smoker | 728 (53.06) | 777 (56.72) | 840 (61.31) | 925 (67.57) | <0.0001 |
| Ex-smoker | 343 (25.00) | 399 (29.12) | 399 (29.12) | 379 (27.68) |  |
| Current smoker | 301 (21.94) | 194 (14.16) | 131 (9.56) | 65 (4.75) |  |
| **Household income** | | | | | |
| Low income ($AUD 1 – 36,999) | 127 (11.07) | 89 (7.41) | 73 (5.94) | 65 (5.33) | <0.0001 |
| Medium income ($AUD 37,000– 77,999) | 374 (32.61) | 371 (30.89) | 341 (27.77) | 348 (28.55) |  |
| High income ($AUD > 77,999) | 646 (56.32) | 741 (61.70) | 814 (66.29) | 806 (66.12) |  |
| **Highest qualification completed** | | | | | |
| No formal qualification or year 10/12 equiv. | 460 (34.51) | 321 (23.97) | 276 (20.41) | 203 (15.12) | <0.0001 |
| Trade/ apprenticeship | 49 (3.68) | 41 (3.06) | 36 (2.66) | 29 (2.16) |  |
| Certificate/ diploma | 360 (27.01) | 371 (27.71) | 312 (23.08) | 273 (20.33) |  |
| Degree or higher | 464 (34.81) | 606 (45.26) | 728 (53.85) | 838 (62.40) |  |
| **Marital status** | | | | | |
| Married/ De Facto | 1,194 (87.28) | 1,241 (90.92) | 1,281 (93.64) | 1.298 (94.61) | <0.0001 |
| Separated/ divorced | 92 (6.73) | 80 (5.86) | 54 (3.95) | 40 (2.92) |  |
| Widowed | 4 (0.29) | 2 (0.15) | 2 (0.15) | 3 (0.22) |  |
| Never married | 78 (5.70) | 42 (3.08) | 31 (2.27) | 31 (2.26) |  |
| **Number of children** | | | | | |
| No children | 134 (9.80) | 187 (13.65) | 174 (12.72) | 198 (14.46) | <0.0001 |
| 1 | 278 (20.34) | 329 (24.01) | 381 (27.85) | 454 (33.16) |  |
| 2-3 | 858 (62.77) | 802 (58.54) | 750 (54.82) | 668 (48.79) |  |
| >3 | 97 (7.10) | 52 (3.80) | 63 (4.61) | 49 (3.58) |  |
| **PCOS status** | | | | | |
| No PCOS | 1,248 (91.23) | 1,228 (89.83) | 1,231 (90.38) | 1,221 (89.32) | 0.38 |
| PCOS | 120 (8.77) | 139 (10.17) | 131 (9.62) | 146 (10.68) |  |
| **Endometriosis status** | | | | | |
| No endometriosis | 1,218 (95.68) | 1,211 (95.88) | 1,193 (95.14) | 1,188 (94.96) | 0.65 |
| Endometriosis | 55 (4.32) | 52 (4.12) | 61 (4.86) | 63 (5.04) |  |

Continuous data are expressed as median (Interquartile range) or mean ± standard deviation, and categorical variables are expressed as frequency (within-column relative frequencies). Numbers may vary due to missing baseline values. Data were analysed with an analysis of variance (ANOVA) or Kruskal-Wallis with post-hoc Dunn’s test for multiple comparisons for normal or non-normal data, respectively. χ^2^ tests were used for categorical variables. P represents the result from the ANOVA, Kruskal-Wallis test, or χ^2^ test, with two-tailed p<0.05 considered statistically significant. Only significant findings for pairwise comparisons with Q1 are displayed for the purposes of simplicity. *P<0.05 for Q4 vs Q1. **P<0.01 for Q4 vs Q1. ***P<0.0001 for Q2/Q3/Q4 vs Q1. **BMI,** body mass index**; IVF,** in-vitro fertilisation; **kJ,** kilojoule; **PCOS,** polycystic ovary syndrome; **Q,** quartile.

**Table S3**. Baseline characteristics of women at survey 5 (1973-1978) stratified by quartile for PCA-derived Western-style (A), Mediterranean-style (B) and plant-dominant (C) dietary patterns.

| **(A)** | Western-style dietary pattern | | | | ***p*** |
| --- | --- | --- | --- | --- | --- |
|  | **Q1**  **(-4.48 - -1.28)** | **Q2**  **(-1.28 - -0.28)** | **Q3**  **(-0.28 - 0.96)** | **Q4**  **(0.96 - 10.67)** |  |
| ***n*** | **1,373** | **1,372** | **1,372** | **1,372** |  |
| Age (years) | 33.84 ± 1.46 | 33.83 ± 1.47 | 33.78 ± 1.45 | 33.83 ± 1.43 | 0.58 |
| **Anthropometry** |  |  |  |  |  |
| Weight (kg) | 65.0 (58.0-75.0) | 67.0 (60.0-76.0) * | 68.0 (60.0-80.0) ** | 70.0 (61.0-84.0) ** | 0.0001 |
| BMI (kg/m^2^) | 23.8 (21.40-27.50) | 24.10 (21.80-27.80) * | 24.80 (22.0-29.0) ** | 25.40 (22.20-30.10) ** | 0.0001 |
| Waist circumference (cm) | 83.77 ± 12.50 | 85.49 ± 12.30* | 88.36 ± 14.45** | 90.13 ± 15.34** | <0.0001 |
| **Physical activity (metabolic minutes/week)** | | | | | |
| Nil/sedentary (<33.3) | 126 (9.39) | 158 (12.03) | 237 (17.93) | 253 (19.02) | <0.0001 |
| Low (33.3 ≤ x < 500) | 491 (36.59) | 556 (42.35) | 548 (41.45) | 581 (43.68) |  |
| Medium (500 ≤ x < 1000) | 308 (22.95) | 314 (23.91) | 294 (22.24) | 266 (20.00) |  |
| High (1000≤) | 417 (31.07) | 285 (21.71) | 243 (18.38) | 230 (17.29) |  |
| **Total energy** | | | | | |
| Total energy intake (kJ/day) | 4815.83 ± 1223.58 | 6128.09 ± 1158.51** | 7341.94 ± 1254.23** | 9628.85 ± 1926.12** | <0.0001 |
| **Frequency of alcohol consumption** | | | | | |
| Never drinks | 200 (14.58) | 173 (12.63) | 172 (12.58) | 203 (14.83) | 0.13 |
| < Once a month | 338 (24.64) | 342 (24.96) | 365 (26.70) | 373 (27.25) |  |
| < Once a week | 297 (21.65) | 303 (22.12) | 318 (23.26) | 301 (21.99) |  |
| 1 or 2 days/week | 285 (20.77) | 288 (21.02) | 248 (18.14) | 252 (18.41) |  |
| 3/4/5/6 days/week | 223 (16.25) | 243 (17.74) | 231 (16.90) | 203 (14.83) |  |
| Every day | 29 (2.11)0 | 21 (1.53) | 33 (2.41) | 37 (2.70) |  |
| **Smoking classification** | | | | | |
| Never smoker | 850 (61.91) | 875 (63.82) | 808 (58.98) | 737 (53.91) | <0.0001 |
| Ex-smoker | 393 (28.62) | 346 (25.24) | 388 (28.32) | 393 (28.75) |  |
| Current smoker | 130 (9.47) | 150 (10.94) | 174 (12.70) | 237 (17.34) |  |
| **Household income** | | | | | |
| Low income ($AUD 1 – 36,999) | 64 (5.42) | 85 (6.93) | 88 (7.26) | 117 (9.95) | <0.0001 |
| Medium income ($AUD 37,000– 77,999) | 298 (25.23) | 359 (29.28) | 370 (30.53) | 407 (34.61) |  |
| High income ($AUD > 77,999) | 819 (69.35) | 782 (63.78) | 754 (62.21) | 652 (55.44) |  |
| **Highest qualification completed** | | | | | |
| No formal qualification or year 10/12 equiv. | 245 (18.22) | 270 (20.07) | 343 (25.71) | 402 (29.93) | <0.0001 |
| Trade/ apprenticeship | 32 (2.38) | 31 (2.30) | 46 (3.45) | 46 (3.43) |  |
| Certificate/ diploma | 309 (22.97) | 335 (24.91) | 323 (24.21) | 349 (25.99) |  |
| Degree or higher | 759 (56.43) | 709 (52.71) | 622 (46.63) | 546 (40.66) |  |
| **Marital status** | | | | | |
| Married/De Facto | 1241 (90.78) | 1267 (92.62) | 1273 (92.92) | 1233 (90.13) | 0.01 |
| Separated/ divorced | 89 (6.51) | 57 (4.17) | 50 (3.65) | 70 (5.12) |  |
| Widowed | 4 (0.29) | 3 (0.22) | 2 (0.15) | 2 (0.15) |  |
| Never married | 33 (2.41) | 41 (3.00) | 45 (3.28) | 63 (4.61) |  |
| **Number of children** | | | | | |
| No children | 257 (18.80) | 176 (12.85) | 141 (10.31) | 119 (8.69) | <0.0001 |
| 1 | 380 (27.80) | 364 (26.57) | 371 (27.12) | 327 (23.89) |  |
| 2-3 | 690 (50.48) | 778 (56.79) | 793 (57.97) | 817 (59.68) |  |
| >3 | 40 (2.93) | 52 (3.80) | 63 (4.61) | 106 (7.74) |  |
| **PCOS status** | | | | | |
| No PCOS | 1231 (90.18) | 1248 (91.23) | 1238 (90.70) | 1211 (88.65) | 0.13 |
| PCOS | 134 (9.82) | 120 (8.77) | 127 (9.30) | 155 (11.35 |  |
| **Endometriosis status** | | | | | |
| No endometriosis | 1200 (94.79) | 1175 (94.30) | 1215 (96.35) | 1220 (96.21) | 0.03 |
| Endometriosis | 66 (5.21) | 71 (5.70) | 46 (3.65) | 48 (3.79) |  |
| \| **(B)** \| **Mediterranean-style dietary pattern** \| \| \| \| ***p*** \| \| --- \| --- \| --- \| --- \| --- \| --- \| \| **Q1**  **(-4.41 - -1.18)** \| **Q2**  **(-1.18 - -0.25)** \| **Q3**  **(-0.24 - 0.92)** \| **Q4**  **(0.92 - 10.53)** \| \| ***n*** \| **1,373** \| **1,372** \| **1,372** \| **1,372** \| \| Age (years) \| 33.81 ± 1.48 \| 33.80 ± 1.45 \| 33.81 ± 1.46 \| 33.85 ± 1.41 \| 0.79 \| \| **Anthropometry** \|  \|  \|  \|  \|  \| \| Weight (kg) \| 68.0 (60.0-80.0) \| 68.0 (60.0-80.0) \| 68.0 (60.0-80.0) \| 67.0 (60.0-77.0) * \| 0.04 \| \| BMI (kg/m^2^) \| 25.10 (22.10-29.30) \| 24.50 (21.90-28.70) \| 24.50 (21.70-28.40) * \| 23.90 (21.50-27.70) ** \| 0.0001 \| \| Waist circumference (cm) \| 88.41 ± 14.28 \| 87.11 ± 14.20 \| 86.75 ± 13.54* \| 85.46 ± 13.46** \| <0.0001 \| \| **Physical activity (metabolic minutes/week)** \| \| \| \| \| \| \| Nil/sedentary (<33.3) \| 284 (21.43) \| 208 (15.72) \| 149 (11.23) \| 133 (9.98) \| <0.0001 \| \| Low (33.3 ≤ x < 500) \| 598 (45.13) \| 580 (43.84) \| 536 (40.39) \| 462 (34.68) \| \| Medium (500 ≤ x < 1000) \| 243 (18.34) \| 275 (20.79) \| 330 (24.87) \| 334 (25.08) \| \| High (1000≤) \| 200 (15.09) \| 260 (19.65) \| 312 (23.51) \| 403 (30.26) \| \| **Total energy** \| \| \| \| \| \| \| Total energy intake (kJ/day) \| 6113.73 ± 2098.20 \| 6560.67 ± 2003.17 \| 7151.14 ± 2108.17 \| 8088.23 ± 2375.75 \| <0.0001 \| \| **Frequency of alcohol consumption** \| \| \| \| \| \| \| Never drinks \| 208 (15.18) \| 192 (14.01) \| 168 (12.26) \| 180 (13.16) \| <0.0001 \| \| < Once a month \| 434 (31.68) \| 375 (27.37) \| 310 (22.63) \| 299 (21.86) \| \| < Once a week \| 323 (23.58) \| 302 (22.04) \| 312 (22.77) \| 282 (20.61) \| \| 1 or 2 days/week \| 219 (15.99) \| 255 (18.61) \| 312 (22.77) \| 287 (20.98) \| \| 3/4/5/6 days/week \| 164 (11.97) \| 218 (15.91) \| 240 (17.52) \| 278 (20.32) \| \| Every day \| 22 (1.61) \| 28 (2.04) \| 28 (2.04) \| 42 (3.07) \| \| **Smoking classification** \| \| \| \| \| \| \| Never smoker \| 777 (56.72) \| 836 (60.93) \| 839 (61.15) \| 818 (59.84) \| <0.0001 \| \| Ex-smoker \| 337 (24.60) \| 357 (26.02) \| 393 (28.64) \| 433 (31.68) \| \| Current smoker \| 256 (18.69) \| 179 (13.05) \| 140 (10.20) \| 116 (8.49) \| \| **Household income** \| \| \| \| \| \| \| Low income ($AUD 1 – 36,999) \| 111 (9.41) \| 80 (6.66) \| 75 (6.27) \| 88 (7.23) \| <0.0001 \| \| Medium income ($AUD 37,000– 77,999) \| 402 (34.10) \| 358 (29.78) \| 348 (29.07) \| 326 (26.79) \| \| High income ($AUD > 77,999) \| 666 (56.49) \| 764 (63.56) \| 774 (64.66) \| 803 (65.98) \| \| **Highest qualification completed** \| \| \| \| \| \| \| No formal qualification or year 10/12 equiv. \| 454 (33.68) \| 328 (24.39) \| 251 (18.73) \| 227 (17.02) \| <0.0001 \| \| Trade/ apprenticeship \| 43 (3.19) \| 40 (2.97) \| 36 (2.69) \| 36 (2.70) \| \| Certificate/ diploma \| 395 (29.30) \| 344 (25.58) \| 316 (23.58) \| 261 (19.57) \| \| Degree or higher \| 456 (33.83) \| 633 (47.06) \| 737 (55.00) \| 810 (60.72) \| \| **Marital status** \| \| \| \| \| \| \| Married/De Facto \| 1220 (89.18) \| 1252 (91.59) \| 1278 (93.42) \| 1264 (92.26) \| 0.001 \| \| Separated/ divorced \| 84 (6.14) \| 70 (5.12) \| 54 (3.95) \| 58 (4.23) \| \| Widowed \| 2 (0.15) \| 7 (0.51) \| 1 (0.07) \| 1 (0.07) \| \| Never married \| 62 (4.53) \| 38 (2.78) \| 35 (2.56) \| 47 (3.43) \| \| **Number of children** \| \| \| \| \| \| \| No children \| 123 (8.98) \| 158 (11.56) \| 183 (13.38) \| 229 (16.72) \| <0.0001 \| \| 1 \| 324 (23.67) \| 362 (26.48) \| 369 (26.97) \| 387 (28.25) \| \| 2-3 \| 848 (61.94) \| 777 (56.84) \| 757 (55.34) \| 696 (50.80) \| \| >3 \| 74 (5.41) \| 70 (5.12) \| 59 (4.31) \| 58 (4.23) \| \| **PCOS status** \| \| \| \| \| \| \| No PCOS \| 1260 (91.90) \| 1227 (90.02) \| 1236 (90.48) \| 1205 (88.34) \| 0.02 \| \| PCOS \| 111 (8.10) \| 136 (9.98) \| 130 (9.52) \| 159 (11.66) \| \| **Endometriosis status** \| \| \| \| \| \| \| No endometriosis \| 1226 (95.48) \| 1183 (95.63) \| 1200 (95.69) \| 1201 (94.87) \| 0.74 \| \| Endometriosis \| 58 (4.52) \| 54 (4.37) \| 54 (4.31) \| 65 (5.13) \| \| \| **(C)** \| **Plant-dominant dietary pattern** \| \| \| \| ***p*** \| \| --- \| --- \| --- \| --- \| --- \| --- \| \| **Q1**  **(-4.48 - -1.04)** \| **Q2**  **(-1.04 - -0.23)** \| **Q3**  **(-0.23 - 0.81)** \| **Q4**  **(0.81 - 12.50)** \| \| ***n*** \| **1,373** \| **1,372** \| **1,372** \| **1,372** \| \| Age (years) \| 33.88 ± 1.45 \| 33.78 ± 1.45 \| 33.81 ± 1.46 \| 33.80 ± 1.43 \| 0.45 \| \| **Anthropometry** \|  \|  \|  \|  \|  \| \| Weight (kg) \| 65.0 (58.0-76.0) \| 67.0 (60.0-78.0) * \| 68.0 (60.0-80.0) ** \| 70.0 (61.0-82.0) ** \| 0.0001 \| \| BMI (kg/m^2^) \| 24.0 (21.5-27.6) \| 24.2 (21.8-28.1) \| 24.5 (21.9-28.7) \| 25.3 (22.0-29.4) \| 0.0001 \| \| Waist circumference (cm) \| 85.78 ± 14.24 \| 85.85 ± 12.82 \| 87.16 ± 14.17 \| 88.73 ± 14.12** \| 0.002 \| \| **Physical activity (metabolic minutes/week)** \| \| \| \| \| \| \| Nil/sedentary (<33.3) \| 200 (15.04) \| 182 (13.79) \| 176 (13.21) \| 216 (16.30) \| 0.42 \| \| Low (33.3 ≤ x < 500) \| 564 (42.41) \| 528 (40.00) \| 550 (41.29) \| 534 (40.30) \| \| Medium (500 ≤ x < 1000) \| 289 (21.73) \| 304 (23.03) \| 304 (22.82) \| 285 (21.51) \| \| High (1000≤) \| 277 (20.83) \| 306 (23.18) \| 302 (22.67) \| 290 (21.89) \| \| **Total energy** \| \| \| \| \| \| \| Total energy intake (kJ/day) \| 6127.86 ± 2081.96 \| 6536.67 ± 2055.20** \| 7177.25 ± 2143.25** \| 8071.97 ± 2319.83** \| <0.0001 \| \| **Frequency of alcohol consumption** \| \| \| \| \| \| \| Never drinks \| 175 (12.79) \| 170 (12.40) \| 184 (13.41) \| 219 (16.02) \| <0.0001 \| \| < Once a month \| 293 (21.42) \| 319 (23.27) \| 354 (25.80) \| 452 (33.07) \| \| < Once a week \| 285 (20.83) \| 315 (22.98) \| 321 (23.40) \| 298 (21.80) \| \| 1 or 2 days/week \| 293 (21.42) \| 289 (21.08) \| 273 (19.90) \| 218 (15.95) \| \| 3/4/5/6 days/week \| 283 (20.69) \| 250 (18.23) \| 212 (15.45) \| 155 (11.34) \| \| Every day \| 39 (2.85) \| 28 (2.04) \| 28 (2.04) \| 25 (1.83) \| \| Smoking classification \| \| \| \| \| \| \| Never smoker \| 779 (56.82) \| 823 (60.03) \| 846 (61.71) \| 822 (60.09) \| 0.06 \| \| Ex-smoker \| 389 (28.37) \| 392 (28.59) \| 367 (26.77) \| 372 (27.19) \| \| Current smoker \| 203 (14.81) \| 156 (11.38) \| 158 (11.52) \| 174 (12.72) \| \| **Household income** \| \| \| \| \| \| \| Low income ($AUD 1 – 36,999) \| 90 (7.71) \| 78 (6.43) \| 90 (7.39) \| 96 (8.02) \| <0.0001 \| \| Medium income ($AUD 37,000– 77,999) \| 283 (24.25) \| 325 (26.79) \| 370 (30.38) \| 456 (38.10) \| \| High income ($AUD > 77,999) \| 794 (68.04) \| 810 (66.78) \| 758 (62.23) \| 645 (53.88) \| \| **Highest qualification completed** \| \| \| \| \| \| \| No formal qualification or year 10/12 equiv. \| 289 (21.63) \| 277 (20.58) \| 309 (22.99) \| 385 (28.71) \| <0.0001 \| \| Trade/ apprenticeship \| 29 (2.17) \| 37 (2.75) \| 42 (3.12) \| 47 (3.50) \| \| Certificate/ diploma \| 310 (23.20) \| 339 (25.19) \| 327 (24.33) \| 340 (25.35) \| \| Degree or higher \| 708 (52.99) \| 693 (51.49) \| 666 (49.55) \| 569 (42.43 \| \| **Marital status** \| \| \| \| \| \| \| Married/De Facto \| 1221 (89.19) \| 1266 (92.48) \| 1271 (92.91) \| 1256 (91.88) \| 0.01 \| \| Separated/ divorced \| 75 (5.48) \| 65 (4.75) \| 54 (3.95) \| 72 (5.27) \| \| Widowed \| 3 (0.22) \| 3 (0.22) \| 3 (0.22) \| 2 (0.15) \| \| Never married \| 70 (5.11) \| 35 (2.56) \| 40 (2.92) \| 37 (2.71) \| \| **Number of children** \| \| \| \| \| \| \| No children \| 232 (16.96) \| 204 (14.91) \| 139 (10.14) \| 118 (8.63) \| <0.0001 \| \| 1 \| 386 (28.22) \| 341 (24.93) \| 378 (27.57) \| 337 (24.65) \| \| 2-3 \| 706 (51.61) \| 774 (56.58) \| 786 (57.33) \| 812 (59.40) \| \| >3 \| 44 (3.22) \| 49 (3.58) \| 68 (4.96) \| 100 (7.32) \| \| **PCOS status** \| \| \| \| \| \| \| No PCOS \| 1222 (89.33) \| 1231 (90.12) \| 1233 (90.33) \| 1242 (90.99) \| 0.54 \| \| PCOS \| 146 (10.67) \| 135 (9.88) \| 132 (9.67) \| 123 (9.01) \| \| **Endometriosis status** \| \| \| \| \| \| \| No endometriosis \| 1198 (94.41) \| 1200 (95.77) \| 1191 (95.20) \| 1221 (96.29) \| 0.13 \| \| Endometriosis \| 71 (5.59) \| 53 (4.23) \| 60 (4.80) \| 47 (3.71) \| \| \| \| \| \| \| | | | | | |

Continuous data are expressed as mean ± standard deviation or median (interquartile range), and categorical variables are expressed as frequency (within-column relative frequencies). Numbers may vary due to missing baseline values. Data were analysed with an analysis of variance (ANOVA) or Kruskal-Wallis with post-hoc Dunn’s test for multiple comparisons for normal or non-normal data, respectively. χ^2^ tests were used for categorical variables. P represents the result from the ANOVA, Kruskal-Wallis test, or χ^2^ test, with two-tailed p<0.05 considered statistically significant. *P<0.05 and **P<0.0001 for Q2/Q3/Q4 vs Q1. **BMI,** body mass index**; IVF,** in-vitro fertilisation; **kJ,** kilojoule; **PCOS,** polycystic ovary syndrome; **Q,** quartile.

**Table S4**. Daily food items and nutrients of women from the Australian Longitudinal Women's Health Study at survey 5 (1973-1978), stratified by quartile of energy-adjusted dietary inflammatory index (E-DII).

| **Item** | **E-DII** | | | | |
| --- | --- | --- | --- | --- | --- |
|  | **Q1**  **(-4.41 – -0.98)** | **Q2**  **(-0.98 – -0.06)** | **Q3**  **(-0.06 – 0.91)** | **Q4**  **(0.91 – 4.25)** | ***p*** |
| **n** | **1,373** | **1,372** | **1,372** | **1,372** |  |
| Total energy (kJ/day) | 6660.74 ± 2450.75 | 6754.68 ± 2 246.36 | 7000.06 ± 2225.11 | 7497.88 ± 2064.76 | <0.0001 |
| Carbohydrates (% energy) | 43.95 ± 6.23 | 42.49 ± 5.10 | 41.27 ± 4.90 | 39.92 ± 5.50 | <0.0001 |
| Protein (% energy) | 20.97 ± 3.36 | 20.72 ± 2.97 | 20.32 ± 2.92 | 19.83 ± 3.29 | <0.0001 |
| Total fat (% energy) | 35.28 ± 5.32 | 37.07 ± 4.35 | 38.77 ± 3.91 | 40.74 ± 3.88 | <0.0001 |
| Saturated fat (% energy) | 13.39 ± 2.53 | 15.50 ± 2.38 | 16.84 ± 2.43 | 18.35 ± 2.47 | <0.0001 |
| MUFA (% energy) | 12.78 ± 2.41 | 13.18 ± 1.87 | 13.61 ± 1.66 | 14.25 ± 1.70 | <0.0001 |
| PUFA (% energy) | 5.82 ± 2.01 | 5.06 ± 1.47 | 4.96 ± 1.45 | 4.74 ± 1.29 | <0.0001 |
| Cholesterol (mg/1000kJ) | 38.13 ± 14.41 | 38.83 ± 10.93 | 40.41 ± 10.50 | 41.75 ± 10.75 | <0.0001 |
| Dietary fibre (g/1000kJ) | 3.56 ± 0.69 | 3.00 ± 0.43 | 2.64 ± 0.39 | 2.16 ± 0.37 | <0.0001 |
| Sugars (% energy) | 1.75 ± 2.30 | 1.98 ± 2.40 | 2.37 ± 2.55 | 3.18 ± 3.36 | <0.0001 |
| Magnesium (mg/1000kJ) | 44.49 ± 6.42 | 39.79 ± 5.03 | 36.84 ± 4.51 | 32.56 ± 4.17 | <0.0001 |
| Potassium (mg/1000kJ) | 435.78 ± 65.26 | 395.20 ± 49.59 | 365.10 ± 45.43 | 327.19 ± 47.31 | <0.0001 |
| Calcium (mg/1000kJ) | 139.22 ± 40.64 | 132.58 ± 36.84 | 128.09 ± 36.49 | 118.50 ± 34.31 | <0.0001 |
| Iron (mg/1000kJ) | 1.95 ± 0.39 | 1.78 ± 0.33 | 1.66 ± 0.27 | 1.49 ± 0.24 | <0.0001 |
| Zinc (mg/1000kJ) | 1.64 ± 0.28 | 1.64 ± 0.24 | 1.60 ± 0.24 | 1.55 ± 0.29 | <0.0001 |
| Vitamin C (mg/1000kJ) | 20.49 ± 7.52 | 16.68 ± 6.38 | 13.83 ± 5.50 | 11.08 ± 5.16 | <0.0001 |
| Vitamin E (mg/1000kJ) | 1.01 ± 0.23 | 0.83 ± 0.13 | 0.77 ± 0.11 | 0.70 ± 0.11 | <0.0001 |
| Retinol (µg/1000kJ) | 40.58 ± 14.68 | 44.91 ± 13.95 | 49.69 ± 14.61 | 53.3 ± 15.45 | <0.0001 |
| Sodium (mg/1000kJ) | 325.34 ± 50.53 | 333.24 ± 45.62 | 335.31 ± 45.62 | 334.42 ± 46.81 | <0.0001 |
| **Whole food items (g/day)** | | | | | |
| Wholegrains | 108.16 ± 82.32 | 106.71 ± 81.58 | 98.75 ± 78.72 | 69.79 ± 65.66 | <0.0001 |
| Refined grains | 83.61 ± 59.27 | 97.49 ± 66.92 | 106.13 ± 71.01 | 124.51 ± 76.09 | <0.0001 |
| Nuts/ nut spread | 6.36 ± 8.39 | 4.78 ± 6.05 | 4.37 ± 5.63 | 3.58 ± 4.94 | <0.0001 |
| Red meat | 50.75 ± 38.84 | 67.52 ± 43.28 | 72.81 ± 48.06 | 83.92 ± 61.82 | <0.0001 |
| Processed meat | 15.95 ± 14.69 | 23.53 ± 17.99 | 26.27 ± 19.05 | 33.61 ± 26.56 | <0.0001 |
| Poultry | 25.15 ± 18.49 | 30.68 ± 20.74 | 32.46 ± 20.63 | 36.44 ± 27.39 | <0.0001 |
| Take away foods | 29.17 ± 21.39 | 38.90 ± 25.73 | 46.33 ± 31.58 | 57.11 ± 39.87 | <0.0001 |
| Fried fish | 3.58 ± 5.75 | 5.36 ± 7.57 | 5.2 ± 6.11 | 7.22 ± 10.70 | <0.0001 |
| Processed fish | 13.24 ± 17.04 | 10.99 ± 14.85 | 10.50 ± 17.56 | 9.08 ± 13.80 | <0.0001 |
| Fish | 17.16 ± 18.47 | 15.34 ± 15.71 | 14.36 ± 16.48 | 14.30 ± 17.46 | <0.0001 |
| Yellow or red vegetables | 34.72 ± 19.70 | 31.55 ± 19.48 | 26.68 ± 16.34 | 20.08 ± 14.22 | <0.0001 |
| Other vegetables* | 27.84 ± 15.10 | 23.36 ± 12.51 | 20.46 ± 11.09 | 17.11 ± 10.61 | <0.0001 |
| Legumes | 32.25 ± 20.70 | 29.55 ± 19.42 | 25.42 ± 17.18 | 20.25 ± 15.67 | <0.0001 |
| Cruciferous vegetables | 31.06 ± 21.81 | 26.88 ± 19.22 | 21.8 ± 16.84 | 16.57 ± 14.42 | <0.0001 |
| Leafy green vegetables | 17.58 ± 12.84 | 13.54 ± 9.36 | 11.11 ± 7.41 | 8.52 ± 6.94 | <0.0001 |
| Tomato | 12.73 ± 12.01 | 10.84 ± 9.42 | 10.05 ± 8.55 | 8.27 ± 7.68 | <0.0001 |
| Fresh fruit | 218.48 ± 121.62 | 184.37 ± 108.90 | 149.26 ± 93.76 | 108.54 ± 74.28 | <0.0001 |
| Canned fruit | 15.07 ± 31.77 | 13.59 ± 31.46 | 11.73 ± 23.97 | 9.33 ± 18.39 | <0.0001 |
| Cakes, biscuits, and sweet pastries | 25.97 ± 21.60 | 36.41 ± 29.09 | 44.22 ± 33.10 | 55.19 ± 43.33 | <0.0001 |
| Low fat dairy | 195.14 ± 169.94 | 201.04 ± 180.27 | 180.27 ± 183.47 | 127.82 ± 176.73 | <0.0001 |
| Full fat dairy | 109.55 ± 110.63 | 159.24 ± 153.52 | 196.15 ± 179.70 | 258.01 ± 218.03 | <0.0001 |
| Soya | 51.96 ± 116.52 | 11.56 ± 51.34 | 7.33 ± 36.66 | 2.14 ± 19.10 | <0.0001 |
| Confectionary | 8.37 ± 9.88 | 12.04 ± 12.98 | 13.80 ± 14.51 | 18.55 ± 20.59 | <0.0001 |
| Potatoes | 23.42 ± 25.34 | 28.59 ± 29.20 | 26.29 ± 26.34 | 24.06 ± 27.00 | <0.0001 |
| Eggs | 16.05 ± 12.78 | 14.79 ± 11.87 | 16.09 ± 12.66 | 16.03 ± 12.87 | 0.0147 |
| Alcohol | 109.96 ± 185.55 | 102.07 ± 165.17 | 101.53 ± 192.59 | 93.40 ± 195.52 | 0.1388 |

Data are expressed as means ± standard deviations. Data were analysed using analysis of variance (ANOVA). Two-tailed p<0.05 was considered statistically significant. *Other vegetables category contains beetroot, celery, mushroom, onion, cucumber, and bean sprouts. **kJ,** kilojoule; **Q**, quartile.

**Table S5**. Daily food items and nutrients of women from the Australian Longitudinal Women's Health Study at survey 5 (1973-1978), stratified by quartile of dietary guideline index (DGI).

| **Item** | **Dietary Guideline index** | | | |  |
| --- | --- | --- | --- | --- | --- |
|  | **Q1**  **(38.37-79.44)** | **Q2**  **(79.45-87.70)** | **Q3**  **(87.70-95.17)** | **Q4**  **(95.18-122.15)** | ***p*** |
| **n** | **1,373** | **1,372** | **1,372** | **1,372** |  |
| Total energy intake (kJ/day) | 6660.74 ± 2450.75 | 6754.68 ± 2246.36 | 7000.06 ± 2225.12 | 7497.88 ± 2064.76 | <0.0001 |
| Carbohydrates (% energy) | 40.42 ± 6.02 | 41.55 ± 5.48 | 42.08 ± 5.52 | 43.58 ± 5.10 | <0.0001 |
| Protein (% energy) | 19.79 ± 3.43 | 20.31 ± 3.01 | 20.68 ± 3.11 | 21.06 ± 2.97 | <0.0001 |
| Total fat (% energy) | 40.26 ± 4.36 | 38.50 ± 4.56 | 37.55 ± 4.70 | 35.56 ± 4.54 | <0.0001 |
| Saturated fat (% energy) | 17.62 ± 2.80 | 16.36 ± 2.88 | 15.85 ± 2.92 | 14.25 ± 2.62 | <0.0001 |
| MUFA (% energy) | 14.24 ± 1.91 | 13.66 ± 1.89 | 13.28 ± 1.96 | 12.62 ± 1.93 | <0.0001 |
| PUFA (% energy) | 4.99 ± 1.54 | 5.10 ± 1.57 | 5.08 ± 1.56 | 5.42 ± 1.82 | <0.0001 |
| Cholesterol (mg/1000kJ) | 41.56 ± 12.79 | 39.98 ± 11.72 | 40.32 ± 11.93 | 37.25 ± 10.38 | <0.0001 |
| Dietary fibre (g/1000kJ) | 2.42 ± 0.60 | 2.77 ± 0.63 | 2.92 ± 0.65 | 3.24 ± 0.70 | <0.0001 |
| Sugars (% energy) | 3.37 ± 3.55 | 2.42 ± 2.66 | 2.00 ± 2.26 | 1.49 ± 1.83 | <0.0001 |
| Magnesium (mg/1000kJ) | 33.58 ± 5.37 | 37.42 ± 5.65 | 39.54 ± 5.60 | 43.16 ± 6.30 | <0.0001 |
| Potassium (mg/1000kJ) | 351.01 ± 63.49 | 374.95 ± 63.94 | 389.24 ± 62.18 | 408.13 ± 60.4 | <0.0001 |
| Calcium (mg/1000kJ) | 114.56 ± 33.84 | 126.57 ± 37.34 | 134.99 ± 37.62 | 142.28 ± 36.94 | <0.0001 |
| Iron (mg/1000kJ) | 1.54 ± 0.30 | 1.68 ± 0.31 | 1.75 ± 0.33 | 1.90 ± 0.36 | <0.0001 |
| Zinc (mg/1000kJ) | 1.55 ± 0.30 | 1.59 ± 0.26 | 1.62 ± 0.25 | 1.66 ± 0.26 | <0.0001 |
| Vitamin C (mg/1000kJ) | 14.75 ± 7.29 | 15.49 ± 7.40 | 15.69 ± 7.05 | 16.16 ± 6.64 | <0.0001 |
| Vitamin E (mg/1000kJ) | 0.78 ± 0.16 | 0.82 ± 0.18 | 0.83 ± 0.18 | 0.89 ± 0.22 | <0.0001 |
| Retinol (µg/1000kJ) | 51.42 ± 15.50 | 47.93 ± 15.62 | 47.34 ± 15.28 | 41.80 ± 13.76 | <0.0001 |
| Sodium (mg/1000kJ) | 330.10 ± 50.90 | 334.57 ± 49.66 | 331.72 ± 44.97 | 331.91 ± 43.37 | 0.0973 |
| **Whole food items (g/day)** | | | | | |
| Wholegrains | 51.67 ± 64.61 | 83.95 ± 66.76 | 104.91 ± 69.17 | 142.93 ± 84.19 | <0.0001 |
| Refined grains | 107.32 ± 71.68 | 100.79 ± 71.50 | 97.19 ± 67.16 | 106.42 ± 69.80 | <0.0001 |
| Nuts and nut spread | 3.29 ± 5.22 | 4.27 ± 5.72 | 5.12 ± 6.69 | 6.42 ± 7.57 | <0.0001 |
| Red meat | 69.61 ± 53.83 | 66.89 ± 47.25 | 67.80 ± 49.30 | 70.68 ± 50.16 | 0.1862 |
| Processed meat | 28.88 ± 24.50 | 25.47 ± 20.04 | 23.51 ± 20.06 | 21.40 ± 18.27 | <0.0001 |
| Poultry | 31.25 ± 22.40 | 31.03 ± 20.17 | 31.03 ± 25.03 | 31.43 ± 21.86 | 0.96 |
| Take away foods | 47.82 ± 35.89 | 45.77 ± 33.66 | 40.41 ± 28.94 | 37.50 ± 28.26 | <0.0001 |
| Fried fish | 6.55 ± 10.08 | 5.52 ± 7.83 | 4.75 ± 5.98 | 4.55 ± 6.91 | <0.0001 |
| Processed fish | 9.05 ± 16.16 | 10.03 ± 14.79 | 10.82 ± 14.02 | 13.93 ± 18.13 | <0.0001 |
| Fish | 13.90 ± 19.20 | 13.70 ± 14.18 | 16.15 ± 17.60 | 17.41 ± 16.74 | <0.0001 |
| Yellow or red vegetables | 24.97 ± 17.84 | 26.72 ± 17.83 | 27.82 ± 17.59 | 33.53 ± 19.31 | <0.0001 |
| Other vegetables* | 18.30 ± 12.22 | 20.32 ± 11.71 | 22.91 ± 13.02 | 27.23 ± 13.48 | <0.0001 |
| Legumes | 22.73 ± 17.43 | 24.96 ± 17.74 | 27.68 ± 18.73 | 32.10 ± 20.25 | <0.0001 |
| Cruciferous vegetables | 22.36 ± 19.71 | 23.18 ± 19.21 | 24.02 ± 18.36 | 26.76 ± 18.70 | <0.0001 |
| Leafy green vegetables | 9.89 ± 8.47 | 11.91 ± 9.13 | 13.23 ± 10.01 | 15.72 ± 11.27 | <0.0001 |
| Tomato | 8.41 ± 8.44 | 10.23 ± 9.03 | 10.66 ± 8.83 | 12.59 ± 11.64 | <0.0001 |
| Fresh fruit | 124.61 ± 94.81 | 153.26 ± 98.53 | 174.68 ± 108.55 | 208.15 ± 115.90 | <0.0001 |
| Canned fruit | 8.27 ± 18.20 | 11.40 ± 26.16 | 13.19 ± 30.93 | 16.87 ± 30.23 | <0.0001 |
| Cakes, biscuits, and sweet pastries | 45.45 ± 40.75 | 40.11 ± 32.69 | 39.30 ± 32.70 | 36.91 ± 30.11 | <0.0001 |
| Low fat dairy | 82.87 ± 129.49 | 152.73 ± 161.77 | 198.93 ± 184.97 | 269.82 ± 184.13 | <0.0001 |
| Full fat dairy | 220.56 ± 189.32 | 182.28 ± 185.76 | 179.44 ± 186.69 | 140.58 ± 137.71 | <0.0001 |
| Soya | 4.39 ± 29.32 | 11.38 ± 50.37 | 14.55 ± 59.02 | 42.73 ± 108.44 | <0.0001 |
| Confectionary | 17.11 ± 19.39 | 13.93 ± 15.82 | 11.93 ± 14.12 | 9.78 ± 9.98 | <0.0001 |
| Potatoes | 25.10 ± 26.94 | 25.42 ± 28.54 | 24.89 ± 25.62 | 26.95 ± 27.11 | 0.18 |
| Eggs | 14.91 ± 12.59 | 15.06 ± 12.00 | 16.41 ± 13.04 | 16.58 ± 12.51 | <0.0002 |
| Alcohol | 160.61 ± 263.99 | 119.52 ± 193.01 | 76.94 ± 127.25 | 49.85 ± 82.92 | <0.0001 |

Data are expressed as mean ± standard deviation. Data were analysed with an analysis of variance (ANOVA). Two-tailed p<0.05 was considered statistically significant. **kJ,** kilojoule; **Q,** quartile. *Other vegetables category contains beetroot, celery, mushroom, onion, cucumber, and bean sprouts.

**Table S6**. Sensitivity analysis for multivariable associations between dietary inflammatory index, dietary guideline index, and principal component analysis-derived pattern scores and self-reported fertility problems.

|  | **Model 1** | | | | | | | **Model 2** | | | | | | | **Model 3** | | | | | | | | **Model 4** | | | | |
| --- | --- | --- | --- | --- | --- | --- | --- | --- | --- | --- | --- | --- | --- | --- | --- | --- | --- | --- | --- | --- | --- | --- | --- | --- | --- | --- | --- |
|  | **n** | | **aOR** | | **95% CI** | ***p*** | | **n** | | **aOR** | | **95% CI** | ***p*** | | **n** | | **aOR** | | **95% CI** | ***p*** | | | **n** | **aOR** | **95% CI** | ***p*** |  |
| **E-DII Overall** | 4,538 | 1.13 | | 1.07, 1.21 | | | **<0.0001** | 4,494 | 1.13 | | 1.07, 1.20 | | | **<0.0001** | 4,475 | 1.14 | | 1.07, 1.21 | | | | **<0.0001** | 4,136 | 1.13 | 1.07, 1.20 | **<0.0001** |  |
| **E-DII Quartiles** | | | | | | | | | | | | | | | | | | | | | | |  |  |  |  | |
| Q1 (-3.29 - 0.50) | 1,142 | *reference* | | | | | | 1,134 | *reference* | | | | | | 1,127 |  | | *reference* | | |  | | 1,038 | *reference* | | | |
| Q2 (0.50 - 1.69) | 1,153 | 1.12 | | 0.91, 1.39 | | | 0.28 | 1,141 | 1.11 | | 0.90, 1.37 | | | 0.35 | 1,137 | 1.13 | | 0.91, 1.41 | | | | 0.27 | 1,055 | 1.16 | 0.93, 1.46 | 0.19 |  |
| Q3 (1.69 - 2.65) | 1,155 | 1.43 | | 1.16, 1.76 | | | **0.001** | 1,147 | 1.41 | | 1.14, 1.74 | | | **0.001** | 1,143 | 1.49 | | 1.20, 1.85 | | | | **<0.0001** | 1,043 | 1.48 | 1.18, 1.86 | **0.001** |  |
| Q4 (2.65 - 4.44) | 1,088 | 1.56 | | 1.26, 1.93 | | | **<0.0001** | 1,072 | 1.55 | | 1.25, 1.92 | | | **<0.0001** | 1,068 | 1.58 | | 1.26, 1.98 | | | | **<0.0001** | 1,000 | 1.60 | 1.27, 2.01 | **<0.0001** |  |
| **DGI Overall** | 4,538 | 0.99 | | 0.99, 1.00 | | | 0.06 | 4,494 | 0.99 | | 0.99, 1.00 | | | 0.08 | 4,475 | 0.99 | | 0.98, 0.99 | | | | **0.03** | 4,136 | 0.99 | 0.99, 1.00 | 0.09 |  |
| **DGI Quartiles** |  |  | |  | | |  |  |  | | | | | |  |  | | | | | | |  |  |  |  |  |
| Q1 (38.37 - 79.44) | 1,073 | *reference* | | | | | | 1,053 | *reference* | | | | | | 1,052 | *reference* | | | | | | | 982 | *reference* | | | |
| Q2 (79.45 - 87.70) | 1,131 | 0.98 | | 0.80, 1.21 | | | 0.84 | 1,120 | 0.96 | | 0.78, 1.18 | | | 0.709 | 1,114 | 0.96 | | 0.78, 1.20 | | | | 0.60 | 1,035 | 0.97 | 0.78, 1.21 | 0.79 |  |
| Q3 (87.70 - 95.17) | 1,162 | 0.96 | | 0.79, 1.20 | | | 0.80 | 1,157 | 0.97 | | 0.79, 1.20 | | | 0.81 | 1,149 | 0.97 | | 0.78, 1.21 | | | | 0.69 | 1,057 | 0.96 | 0.77, 1.21 | 0.75 |  |
| Q4 (95.18 - 122.15) | 1,172 | 0.78 | | 0.62, 0.97 | | | **0.03** | 1,164 | 0.78 | | 0.63, 0.97 | | | **0.03** | 1,160 | 0.76 | | 0.60, 0.95 | | | | **0.01** | 1,062 | 0.77 | 0.61, 0.97 | **0.03** |  |
| **Western style** | 4,538 | 1.03 | | 0.99, 1.08 | | | 0.12 | 4,494 | 1.02 | | 0.98, 1.07 | | | 0.32 | 4,475 | 1.02 | | 0.97, 1.06 | | | | 0.41 | 4,136 | 1.03 | 0.99, 1.08 | 0.13 |  |
| **Quartiles** |  |  | |  | | |  |  |  | |  | | |  |  |  | |  | | | |  |  |  |  |  |  |
| Q1 (-4.48 - -1.28) | 1,135 |  | | *reference* | | |  | 1,124 |  | | *reference* | | |  | 1,119 |  | | *reference* | | | |  | 1,040 | *reference* | | |  |
| Q2 (-1.28 - -0.28) | 1,156 | 1.16 | | 0.94, 1.42 | | | 0.16 | 1,147 | 1.12 | | 0.91, 1.38 | | | 0.28 | 1,143 | 1.17 | | 0.94, 1.44 | | | | 0.16 | 1,041 | 1.11 | 0.89, 1.39 | 0.34 |  |
| Q3 (-0.28 - 0.96) | 1,139 | 1.08 | | 0.88, 1.34 | | | 0.45 | 1,127 | 1.04 | | 0.84, 1.28 | | | 0.75 | 1,123 | 1.08 | | 0.87, 1.34 | | | | 0.50 | 1,043 | 1.10 | 0.88, 1.37 | 0.41 |  |
| Q4 (0.96 - 10.67) | 1,108 | 1.22 | | 0.99, 1.51 | | | 0.06 | 1,096 | 1.16 | | 0.94, 1.43 | | | 0.17 | 1,090 | 1.15 | | 0.92, 1.43 | | | | 0.22 | 1,012 | 1.22 | 0.97, 1.53 | 0.08 |  |
| **Mediterranean style** | 4,538 | 0.92 | | 0.88, 0.96 | | | **0.001** | 4,494 | 0.92 | | 0.88, 0.96 | | | **<0.0001** | 4,475 | 0.89 | | 0.85, 0.94 | | | | **<0.0001** | 4,136 | 0.92 | 0.87, 0.97 | **0.001** |  |
| **Quartiles** |  |  | |  | | |  |  |  | |  | | |  |  |  | |  | | | |  |  |  |  |  |  |
| Q1 (-4.41 - -1.18) | 1,112 |  | | *reference* | | |  | 1,096 |  | | *reference* | | |  | 1,095 |  | | *reference* | | | |  | 1,032 | *reference* | | |  |
| Q2 (-1.18 - -0.25) | 1,140 | 0.95 | | 0.77, 1.16 | | | 0.60 | 1,125 | 0.92 | | 0.75, 1.13 | | | 0.41 | 1,119 | 0.90 | | 0.73, 1.11 | | | | 0.34 | 1,016 | 0.93 | 0.75, 1.16 | 0.53 |  |
| Q3 (-0.24 - 0.92) | 1,137 | 0.84 | | 0.68, 1.03 | | | 0.10 | 1,131 | 0.81 | | 0.66, 1.00 | | | 0.051 | 1,124 | 0.80 | | 0.64, 0.99 | | | | **0.04** | 1,033 | 0.83 | 0.66, 1.03 | 0.10 |  |
| Q4 (0.92 - -10.53) | 1,149 | 0.70 | | 0.56, 0.86 | | | **0.001** | 1,142 | 0.68 | | 0.55, 0.85 | | | **0.001** | 1,137 | 0.62 | | 0.50, 0.78 | | | | **<0.0001** | 1,055 | 0.70 | 0.56, 0.88 | **0.002** |  |
| **Plant-dominant style** | 4,538 | 0.96 | | 0.92, 1.01 | | | 0.16 | 4,494 | 0.96 | | 0.91, 1.01 | | | 0.13 | 4,475 | 0.97 | | 0.92, 1.02 | | | | 0.22 | 4,136 | 0.96 | 0.91, 1.05 | 0.10 |  |
| **Quartiles** |  |  | |  | | |  |  |  | |  | | |  |  |  | |  | | | |  |  |  |  |  |  |
| Q1 (-4.48 - -1.04) | 1,102 |  | | *reference* | | |  | 1,093 |  | | *reference* | | |  | 1,084 |  | | *reference* | | | |  | 1,013 | *reference* | | |  |
| Q2 (-1.04 - -0.23) | 1,147 | 0.90 | | 0.73, 1.10 | | | 0.29 | 1,135 | 0.88 | | 0.72, 1.08 | | | 0.23 | 1,132 | 0.91 | | 0.74, 1.13 | | | | 0.39 | 1,041 | 0.91 | 0.73, 1.13 | 0.39 |  |
| Q3 (-0.23 - 0.81) | 1,160 | 0.93 | | 0.76, 1.14 | | | 0.50 | 1,149 | 0.92 | | 0.75, 1.13 | | | 0.43 | 1,145 | 0.93 | | 0.75, 1.15 | | | | 0.53 | 1,045 | 0.98 | 0.79, 1.21 | 0.84 |  |
| Q4 (0.81 - 12.50) | 1,129 | 0.80 | | 0.64, 0.98 | | | **0.03** | 1,117 | 0.79 | | 0.64, 0.98 | | | **0.03** | 1,114 | 0.82 | | 0.66, 1.02 | | | | 0.08 | 1,037 | 0.79 | 0.63, 0.99 | 0.04 |  |

Logistic regression was conducted to derive the odds ratios for the associations between energy-adjusted dietary inflammatory index, dietary guideline index (overall and quartiles), or principal component analysis-derived dietary pattern scores (overall and quartiles) with self-reported fertility problems. Q1 was designated as the reference category. Statistical significance was considered p<0.05. **aOR,** adjusted odds ratio**; CI,** confidence interval; **E-DII,** energy-adjusted dietary inflammatory index**; n,** sample size; **OR,** odds ratio; **Q,** quartile.

Model 1 (primary excluding BMI): adjusted for maternal age, number of children, household income, education, marital status, alcohol consumption, smoking, and physical activity.

Model 2 (primary excluding alcohol consumption): adjusted for maternal age, body mass index, marital status, number of children, household income, education, smoking status and physical activity.

Model 3 (primary including PCOS status): adjusted for maternal age, body mass index, number of children, household income, education, marital status, alcohol consumption, smoking, physical activity, and PCOS status.

Model 4 (primary including endometriosis status): adjusted for maternal age, body mass index, number of children, household income, education, marital status, alcohol consumption, smoking, physical activity, and endometriosis status.

**Table S7**. Sensitivity analysis of associations between dietary inflammatory index, dietary guideline index, and principal component analysis-derived dietary pattern scores with self-reported fertility problems, with multiple imputation by chained equations (MICE).

|  | **Adjusted – Imputed*** | | | | |
| --- | --- | --- | --- | --- | --- |
|  | **n** | | **aOR** | **95% CI** | ***p*** |
| **E-DII Overall** | 5,489 | | 1.11 | 1.06, 1.17 | **<0.0001** |
| **E-DII Quartiles** |  | |  |  |  |
| Q1 (-4.41 – -0.98) | 5,489 | | *reference* | | |
| Q2 (-0.98 – -0.06) | 5,489 | | 1.15 | 0.95, 1.39 | 0.164 |
| Q3 (-0.06 – 0.91) | 5,489 | | 1.34 | 1.11, 1.63 | **0.003** |
| Q4 (0.91 – 4.25) | 5,489 | | 1.48 | 1.22, 1.80 | **<0.0001** |
| **DGI Overall** | 5,489 | | 0.99 | 0.98, 0.99 | **0.007** |
| **DGI Quartiles** |  | |  |  |  |
| Q1 (38.37 –79.44) | 1,373 | | *reference* | | |
| Q2 (79.45 – 87.70) | 1,372 | | 0.93 | 0.77, 1.12 | 0.448 |
| Q3 (87.70 – 95.17) | 1,372 | | 0.93 | 0.77, 1.13 | 0.483 |
| Q4 (95.18 – 122.15) | 1,372 | | 0.73 | 0.60, 0.89 | **0.002** |
| **Principal component analysis** | | | | | |
| **Western style dietary pattern** | 5,489 | 1.03 | | 0.99, 1.07 | 0.14 |
| **Quartiles** |  |  | |  |  |
| Q1 (-4.48 - -1.28) | 1,373 |  | | *reference* |  |
| Q2 (-1.28 - -0.28) | 1,372 | 1.23 | | 1.02, 1.48 | **0.03** |
| Q3 (-0.28 - 0.96) | 1,372 | 1.12 | | 0.93, 1.36 | 0.23 |
| Q4 (0.96 - 10.67) | 1,372 | 1.25 | | 1.03, 1.51 | **0.03** |
| **Mediterranean style dietary pattern** | 5,486 | 0.92 | | 0.88, 0.96 | **<0.0001** |
| **Quartiles** |  |  | |  |  |
| Q1 (-4.41 - -1.18) | 1373 |  | | *reference* |  |
| Q2 (-1.18 - -0.25) | 1372 | 0.93 | | 0.77, 1.12 | 0.44 |
| Q3 (-0.24 - 0.92) | 1372 | 0.82 | | 0.68, 0.99 | **0.04** |
| Q4 (0.92 - -10.53) | 1372 | 0.70 | | 0.57, 0.85 | **<0.0001** |
| **Plant-dominant dietary pattern** | 5,489 | 0.97 | | 0.93, 1.02 | 0.23 |
| **Quartiles** |  |  | |  |  |
| Q1 (-4.48 - -1.04) | 1373 |  | | *reference* |  |
| Q2 (-1.04 - -0.23) | 1372 | 0.92 | | 0.77, 1.11 | 0.40 |
| Q3 (-0.23 - 0.81) | 1372 | 0.95 | | 0.79, 1.15 | 0.63 |
| Q4 (0.81 - 12.50) | 1372 | 0.83 | | 0.68, 1.00 | 0.05 |

Logistic regression was conducted to derive the odds ratios for the associations between energy-adjusted dietary inflammatory index, dietary guideline index (overall and quartiles), or principal component analysis-derived dietary pattern scores (overall and quartiles) with self-reported fertility problems. Q1 was designated as the reference category. Analyses were conducted on imputed data using multiple imputation by chained equations (MICE) to account for missing covariate data. Statistical significance was defined as p < 0.05. *Model 1 was adjusted for maternal age, body mass index, number of children, household income, education, marital status, alcohol consumption, smoking, and physical activity. **aOR,** adjusted odds ratio**; CI,** confidence interval; **E-DII,** energy-adjusted dietary inflammatory index**; n,** sample size; **OR,** odds ratio; **Q,** quartile.

**Table S8.** Kaiser-Meyer-Olkin measure of sampling adequacy and Bartlett’s test of Sphericity to determine the validity of the attained factors for exploratory factor analysis.

| **Factor test** | **Outcome** |
| --- | --- |
| **Kaiser-Meyer-Olkin measure of sampling adequacy** | |
| Score | 0.730 |
| **Bartlett’s test of Sphericity** | |
| Chi-square | 28413.055 |
| Degrees of Freedom | 561 |
| p | <0.0001 |
